# Development of extrinsic innervation in the abdominal intestines of human embryos

**DOI:** 10.1111/joa.13230

**Published:** 2020-06-29

**Authors:** Nutmethee Kruepunga, Jill P. J. M. Hikspoors, Cindy J. M. Hülsman, Greet M. C. Mommen, S. Eleonore Köhler, Wouter H. Lamers

**Affiliations:** ^1^ Department of Anatomy & Embryology Maastricht University Maastricht The Netherlands; ^2^ Department of Anatomy Faculty of Science Mahidol University Bangkok Thailand; ^3^ Tytgat Institute for Liver and Intestinal Research Academic Medical Center Amsterdam The Netherlands

**Keywords:** 3D reconstruction, enteric nervous system, neural crest, para‐aortic ganglia, pre‐aortic plexuses, Schwann cell precursor, splanchnic nerves

## Abstract

Compared to the intrinsic enteric nervous system (ENS), development of the extrinsic ENS is poorly documented, even though its presence is easily detectable with histological techniques. We visualised its development in human embryos and foetuses of 4–9.5 weeks post‐fertilisation using Amira 3D‐reconstruction and Cinema 4D‐remodelling software. The extrinsic ENS originated from small, basophilic neural crest cells (NCCs) that migrated to the para‐aortic region and then continued ventrally to the pre‐aortic region, where they formed autonomic pre‐aortic plexuses. From here, nerve fibres extended along the ventral abdominal arteries and finally connected to the intrinsic system. Schwann cell precursors (SCPs), a subgroup of NCCs that migrate on nerve fibres, showed region‐specific differences in differentiation. SCPs developed into scattered chromaffin cells of the adrenal medulla dorsolateral to the coeliac artery (CA) and into more tightly packed chromaffin cells of the para‐aortic bodies ventrolateral to the inferior mesenteric artery (IMA), with reciprocal topographic gradients between both fates. The extrinsic ENS first extended along the CA and then along the superior mesenteric artery (SMA) and IMA 5 days later. Apart from the branch to the caecum, extrinsic nerves did not extend along SMA branches in the herniated parts of the midgut until the gut loops had returned in the abdominal cavity, suggesting a permissive role of the intraperitoneal environment. Accordingly, extrinsic innervation had not yet reached the distal (colonic) loop of the midgut at 9.5 weeks development. Based on intrinsic ENS‐dependent architectural remodelling of the gut layers, extrinsic innervation followed intrinsic innervation 3–4 Carnegie stages later.

## Graphical abstract

The neural crest cells that form the extrinsic enteric nervous system migrate to the para‐ and then the pre‐aortic regions. Concomitantly, the thoracic (left) and lumbar (right) splanchnic nerves extend ventrally to the pre‐aortic plexuses. Pre‐aortic nerve fibres from the coeliac and inferior mesenteric plexus extend further ventrally to connect to the intrinsic nerve fibres inside the gut wall. The herniated portions of the midgut become innervated only after return into the abdominal cavity.

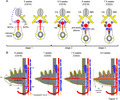

## 1 INTRODUCTION

The enteric nervous system (ENS) is, together with the sympathetic and parasympathetic systems, a component of the autonomic nervous system as defined by Langley (1921). The ENS regulates intestinal motility, secretion and blood flow. The ENS is also known as the ‘little’ or ‘second’ brain because of the large number of enteric neurons (2–6*10^8^ in man (Furness *et al*., 2014) and 1–1.5*10^6^ in mice (Gianino *et al*., 2003)) and their complex network of connections (Gershon, 1999). It consists of intrinsic and extrinsic components. The intrinsic ENS comprises ganglionated plexuses in the wall of the intestine, while the extrinsic ENS is mainly found as catecholaminergic fibres along the arterial trees that perfuse the intestine. The entire ENS originates from neural crest cells ((NCCs); for review, see e.g. (Sasselli *et al*., 2012)). NCCs may well represent the last remaining group of pluripotent cells of the epiblast (Buitrago‐Delgado *et al*., 2015). NCCs make the first of many sequential steps towards differentiation into neuronal or glial cells shortly after delamination from the neural plate (Soldatov *et al*., 2019). This series of sequential binary decisions shows that the fate of NCCs is not pre‐specified, but that their differentiation is accompanied by a progressive restriction of developmental options (Ruhrberg and Schwarz, 2010, Anderson, 1989).

The fates of the head and trunk NCCs differ (Kuratani *et al*., 2018). The cephalic NCCs typically migrate along a dorsolateral pathway underneath the surface epithelium (Serbedzija *et al*., 1992), whereas most truncal NCCs follow a ventrolateral pathway (Serbedzija and McMahon, 1997). The vagal neural crest, which arises adjacent to somites 1–5 in embryonic day (ED) 8.5 mouse embryos (≈ Carnegie stage (CS) 11 in human embryos) (Durbec *et al*., 1996), represents a transitional structure (Kuratani *et al*., 2018): its cranial portion (adjacent to somites 1 and 2) migrates along the dorsolateral route and colonises the emerging vagus nerve alongside the oesophagus and stomach, while its caudal portion (adjacent to somites 3–5) migrates ventrolaterally to populate the emerging cranial portion of the sympathetic trunks and the entire caudal foregut and midgut (Anderson *et al*., 2006; Espinosa‐Medina *et al*., 2014). The minority of NCCs in neck and trunk that follow the dorsolateral pathway expresses the marker c‐KIT, but not the neurotrophin receptor p75^NTR^. The majority of NCCs that follow the ventral pathway, instead, express the reciprocal phenotype (Wilson *et al*., 2004) and pass between the neural tube and the dermomyotomes along the intersomitic blood vessels and then through the cranial portion of the sclerotomes (Serbedzija *et al*., 1990, Ruhrberg and Schwarz, 2010). The earliest group of these cells (ED8.5–9.5 in the mouse, ~CS11‐late) follows a more ventrolateral course towards the para‐aortic space to form the sympathetic trunks, whereas a slightly later group (ED9.5‐ED10.5; CS12‐14) follows a more medial route to the spinal ganglia or become Schwann cells (Serbedzija *et al*., 1990).

Neural crest cells that reside on the spinal nerves often differentiate into Schwann cells, but a subpopulation retains the capacity to differentiate into ganglionic cells. The entire population was referred to as ‘Schwann‐cell precursors’ (SCPs) 30 years ago (Jessen and Mirsky, 1991), but were already recognised by phenotype and position 80 years earlier (Kuntz, 1910). SCPs are found on the vagus nerve (Espinosa‐Medina *et al*., 2014), but also account for as much as 80% of the chromaffin cells of the adrenal medulla (Furlan *et al*., 2017) and para‐aortic bodies (Kastriti *et al*., 2019). Furthermore, up to 5% of the submucosal neurons in the small intestine and ~20% of the submucosal and myenteric neurons in the colon have an SCP origin (Uesaka *et al*., 2015). NCCs that become sympathetic neurons and those that become SCPs and eventually chromaffin cells differ phenotypically in that SCPs express more tyrosine hydroxylase (TH), less cocaine and amphetamine‐regulated transcript (CART) and have a lower proliferative activity than sympathetic neurons well before they arrive at their respective target positions (Chan *et al*., 2016). The decision of cells in sympathetic ganglia or ganglionic plexuses of the gut to differentiate into ganglionic or glial cells depends on upregulation of the expression of transcription factor PHOX2B at ED10.5 (~CS14) (Tiveron *et al*., 1996) and the ensuing downregulation of neural stem‐cell marker SOX10 (Nagashimada *et al*., 2012). NCC migration is largely determined by environmental signals that induce or maintain growth in the NCC population (Young *et al*., 2001, Burns and Thapar, 2006, Vega‐Lopez *et al*., 2017, Lumb *et al*., 2018).

Although the development and fate of the intrinsic ENS has been studied extensively in the last 40 years, the extrinsic ENS has mustered only cursory interest (Uesaka *et al*., 2016). This discrepancy is the more remarkable since at least part of the extrinsic ENS is phenotypically identifiable in standardly stained sections because of the early appearance of the acidophilic staining property of its nerve fibres. Since no recent account of its early topographic development in human embryos is available, we have carried out such a study, using histology as method of identification and three‐dimensional reconstruction as method to visualise developmental changes in architecture and distribution.

## 2 MATERIALS AND METHODS

### 2.1 Embryos

This study was undertaken in accordance with the Dutch regulations for the proper use of human tissue for medical research purposes. Well‐preserved human embryos and foetuses of the historical collections of the Departments of Anatomy and Embryology, Leiden University Medical Centre (LUMC), Leiden, the Academic Medical Centre (AMC), Amsterdam, Radboud University, Nijmegen, the Netherlands, and the University of Göttingen, Germany (Blechschmidt Collection; https://doi.org/10.3249/ugoe‐publ‐2), that were donated for scientific research were studied. In addition, digital images of carefully staged human embryos of the Carnegie collection (Washington DC, USA) were downloaded from the Digitally Reproduced Embryonic Morphology (DREM) project (http://virtualhumanembryo.lsuhsc.edu). A detailed time line of the development of the enteric nervous system in mice can be found in (Sasselli *et al*., 2012; Hao *et al*., 2016).

### 2.2 Image acquisition, 3D reconstruction and visualisation

Human embryos and foetuses between 4 and 9.5 weeks of development were studied. Embryonic development is expressed in Carnegie stages (CS), which, from the 3rd week onward, are virtually identical to Streeter's Developmental Horizons (Streeter, 1951, O'Rahilly and Müller, 1987). CS were converted into estimated embryonic age according to (O'Rahilly and Müller, 2010). A graph relating the Carnegie stages of human embryos to days of development in mice or Hamilton–Hamburger (HH) stages (Hamburger and Hamilton, 1951) in chicken is found in Figure S1. The resolution of the physiological hernia at 9.5 weeks was used as criterion to define foetal ages between 9 and 9.5 weeks of development (Soffers et.al, 2015; Table 1). Serial sections from AMC, LUMC and Radboud embryos were digitised with an Olympus BX51 or BX61 microscope and the Dotslide program (Olympus), whereas those of the Blechschmidt collection were digitised with a Zeiss Axio Scan.Z1 (Carl Zeiss Microscopy). All digital images were converted into greyscale ‘JPEG’ format and loaded into Amira3D (version 6.5; FEI Visualization Sciences Group Europe). The greyscale images were aligned automatically with the least‐squares alignment mode and further adjusted manually for the correct curvature of the embryonic body axis with the help of photographs and magnetic resonance images (MRI) of human embryos of the same stage of development (Pooh *et al*., 2011). Structures of interest were segmented manually and reconstructed three‐dimensionally with the Amira3D program. Because small deformations of individual sections due to histological processing and section stacking introduced a distracting noise into the 3D reconstructions, polygon meshes from all reconstructed materials were exported via ‘vrml export’ from Amira3D to Cinema 4D (MAXON Computer GmbH) and remodelled using the Amira3D model as template. The accuracy of the remodelling process was validated by simultaneous visualisation in Cinema 4D of the Amira3D output and the remodelled Cinema model (Figure S2). The Cinema‐4D models were transferred via ‘wrl export’ to Adobe Acrobat version 9 (http://www.adobe.com) to generate interactive 3D Portable Device Format (PDF) files, which are an easily accessible format for 3D visualisation (Figures [Link], [Link], [Link]). Although we mostly refer in the text to the Figures to relate histology to developing structures, the reader is encouraged to simultaneously inspect the interactive PDFs, because their rotational options (‘live’ images) allow a much better understanding of the complex local topography than the ‘still’ images in the Figures.

**Table 1:** Metadata of human embryos and foetuses that were studied

Stage	Days	Embryo	Fixation	Staining	Plane	Source
CS10	28	S6330	Formalin	Ehrlich's H	Transv	DREM
CS11	29	S6344	Formalin	CA	Transv	DREM
CS12	30	S8943	Zenker's fix	H & E	Transv	DREM
CS13	32	S836	HgCl_2_	CA	Transv	DREM
CS14‐early	33	S2201	Formalin	H & A	Transv	AMC
CS14‐mid	34	S5029	Formalin	H & A	Sagittal	AMC
CS14‐mid	34	S168	Bouin's fix	H & E	Transv	LUMC
CS14‐mid	34	1950‐09‐13		H & E	Sagittal	Göttingen
CS14‐late	35	1958‐12‐22		H & E	Sagittal	Göttingen
CS14‐late	35	1961‐06‐13		H & E	Transv	Göttingen
CS14‐late	35	S6502	Souza's fix	H & E (or + Ag)	Transv	DREM
CS15‐early	36	S721	Zenker's fix	H & E (or + Ag)	Transv	DREM
CS15‐early	36	S79	Formalin	H & E	Transv	LUMC
CS15‐early	36	1945‐10‐26		H & E	Transv	Göttingen
CS15‐early	36	1957‐10‐31		H & E	Transv	Göttingen
CS15‐late	37	S2213	Formalin	H & A	Transv	AMC
CS16	39	S5032	Formalin	H & A	Sagittal	AMC
CS16	39	S6517	Corrosive CH_3_COOH	CA	Transv	DREM
CS16	39	S39	Formalin	H & E	Transv	LUMC
CS17	41	S6520	Corrosive CH_3_COOH	CA (or + Ag)	Transv	DREM
CS18‐early	43	S97	Bouin's fix	H & E	Transv	LUMC
CS18‐late	45	S4430	Corrosive CH_3_COOH	CA	Transv	DREM
CS19	46	S9325	Acetic formalin	Azan & Ag	Transv	DREM
CS20	49	S2025	Bouin's fix	H & A	Transv	AMC
CS20	49	S462	Formalin	CA	Transv	DREM
CS20	49	S34	Formalin & Bouin's fix	H & E	Sagittal	LUMC
CS21	51	S4090	Formalin	CA	Transv	DREM
CS22	53	S48	Formalin	H & E	Transv	LUMC
CS22	54	S983	Formalin	H & E	Transv	DREM
CS23	56	S4141	Formalin	H & A	Transv	AMC
CS23	56	S9226	Formalin	Azan	Transv	DREM
CS23	56	S88	Formalin & Bouin's fix	H or PAS or Azan	Sagittal	RadboudMC
9 weeks	63	S89	Formalin	H & E or Azan	Transv	LUMC
9.5 weeks	67	S57	Formalin	H & E	Transv	LUMC

Note

The estimated post‐fertilisation ages of the embryos are based on (O'Rahilly and Müller, 2010). The additions ‘early’, ‘mid’ and ‘late’ are meant to indicate that, within these stages, the development of the gut and enteric nervous system of ‘late’ embryos was more advanced than that of ‘early’ embryos.

The corresponding age was chosen from the range of developmental days attributed to that stage (O'Rahilly and Müller, 2010).

CS14 in particular is noted for its remarkable number of developmental events.

Abbreviations: AC, alum cochineal (i.e. carmine); AMC, Academic Medical Centre; CS, Carnegie stage; DREM, Carnegie collection from the Digitally Reproduced Embryonic Morphology project; Göttingen, Department of Anatomy and Embryology, Göttingen; H&A, haematoxylin and azophloxine; H&E haematoxylin and eosin; LUMC, Leiden University Medical Centre; PAS, periodic acid–Schiff stain; RadboudMC: Radboud Medical Centre.

### 2.3 Terminology

We categorised the nerve fibres innervating the gut into intrinsic and extrinsic fibres. The well‐studied intrinsic nerve fibres are located in the intestinal wall, whereas the less‐studied extrinsic nerve fibres have their origin outside the gut and reach the gut wall, as we show, predominantly by following the peripheral branches of the intestinal arteries (Uesaka *et al*., 2016).

Intestinal development in avian embryos (Southwell, 2006) proceeds in a similar fashion as in mammalian embryos (Soffers *et al*, 2015), with the midgut or primary loop extending into the coelom of the umbilical cord. In agreement, the umbilical ‘hernia’ contains only the midgut in both vertebrate classes. The main differences appear to be the formation in birds of only a single (duodeno‐jejunal) rather than 4 secondary loops and 2 rather than 1 caecal diverticula. However, the nomenclature used to identify the respective parts of the embryonic gut is not the same and at times confusing. In avian and mammalian embryos, the cells of the vagal neural crest colonise both the ‘pre‐umbilical’ and ‘post‐umbilical’ parts of the gut, whereas sacral NCCs only colonise the ‘post‐umbilical’ gut in a caudocranial direction (Le Douarin & Teillet, 1973; Burns & Le Douarin, 1998; Anderson et al., 2006). The sacral neural crest begins distal to somite 24 in mice (Dong *et al*., 2006) and somite 28 in chicken (Le Douarin & Teillet, 1973), which correspond to vertebral segment L1‐2 in both birds and mammals. In mammalian embryos, the part of the gut colonised by sacral NCCs is often referred to as hindgut (Young et al., 2001; Wang & Chan, 2011) and corresponds with the distal loop of the embryonic midgut and hindgut ‘proper’ in our descriptions. The autonomic innervation of the embryonic hindgut is reported in the accompanying study.

## 3 RESULTS

### 3.1 Early sources of peripheral autonomic ganglionic cells

**Figure 1:**
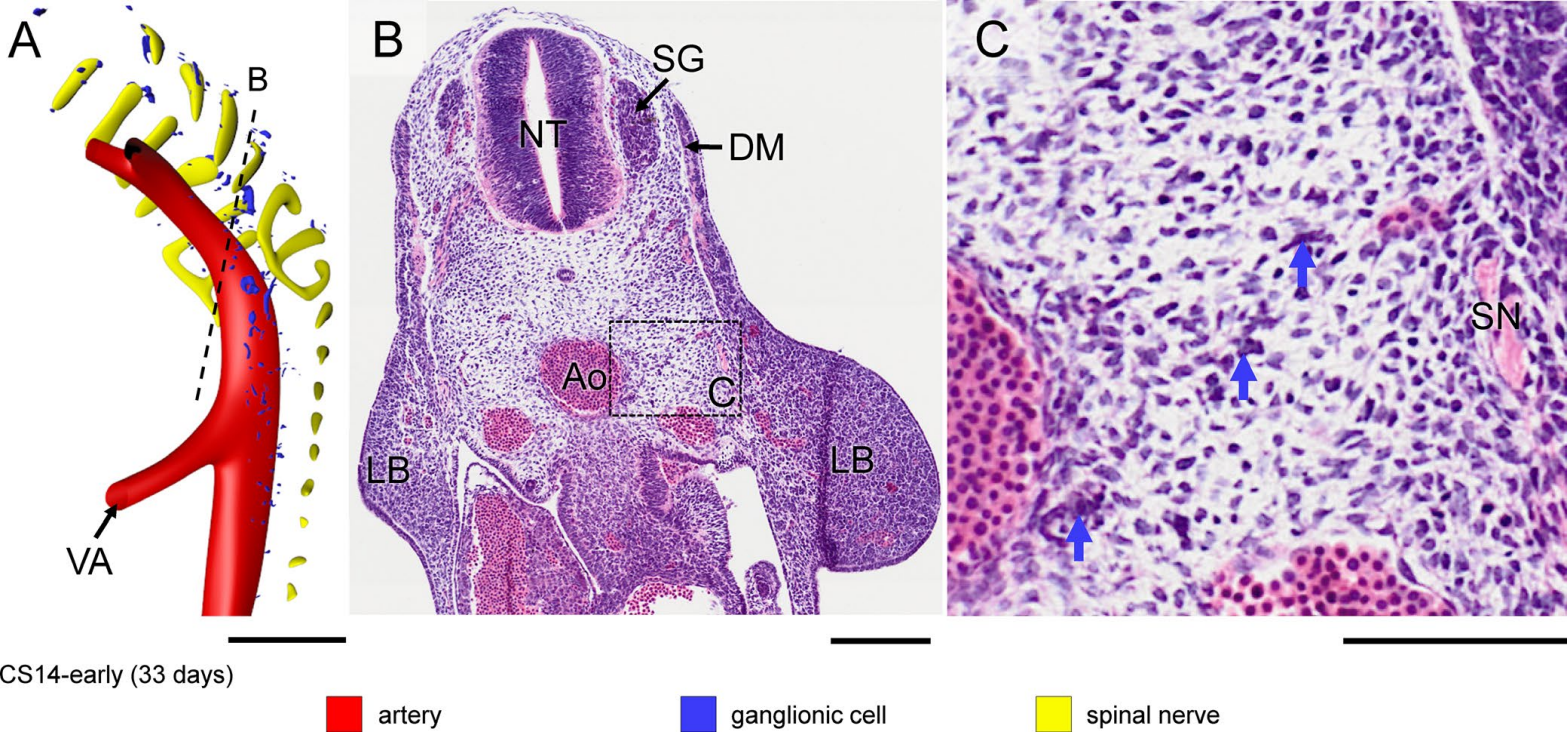
Appearance of neural crest‐cell precursors of ganglionic cells in a CS14‐early embryo (~33 days). Panel (a) shows the distribution of ganglionic precursor cells dorsal and lateral to the dorsal aorta (see also Figure S3A). The ventral roots of the spinal nerves are coded yellow. Panels (b and c) show a section indicated by the dotted line in panel (a) and a magnified view of the rectangle in panel (b). Intensely staining basophilic cells (blue arrows in panel (c)) represent ganglionic precursor cells between spinal nerve (SN) and aorta (Ao) in panel (c). Bars = 200 µm [Colour figure can be viewed at wileyonlinelibrary.com]

**Figure 2:**
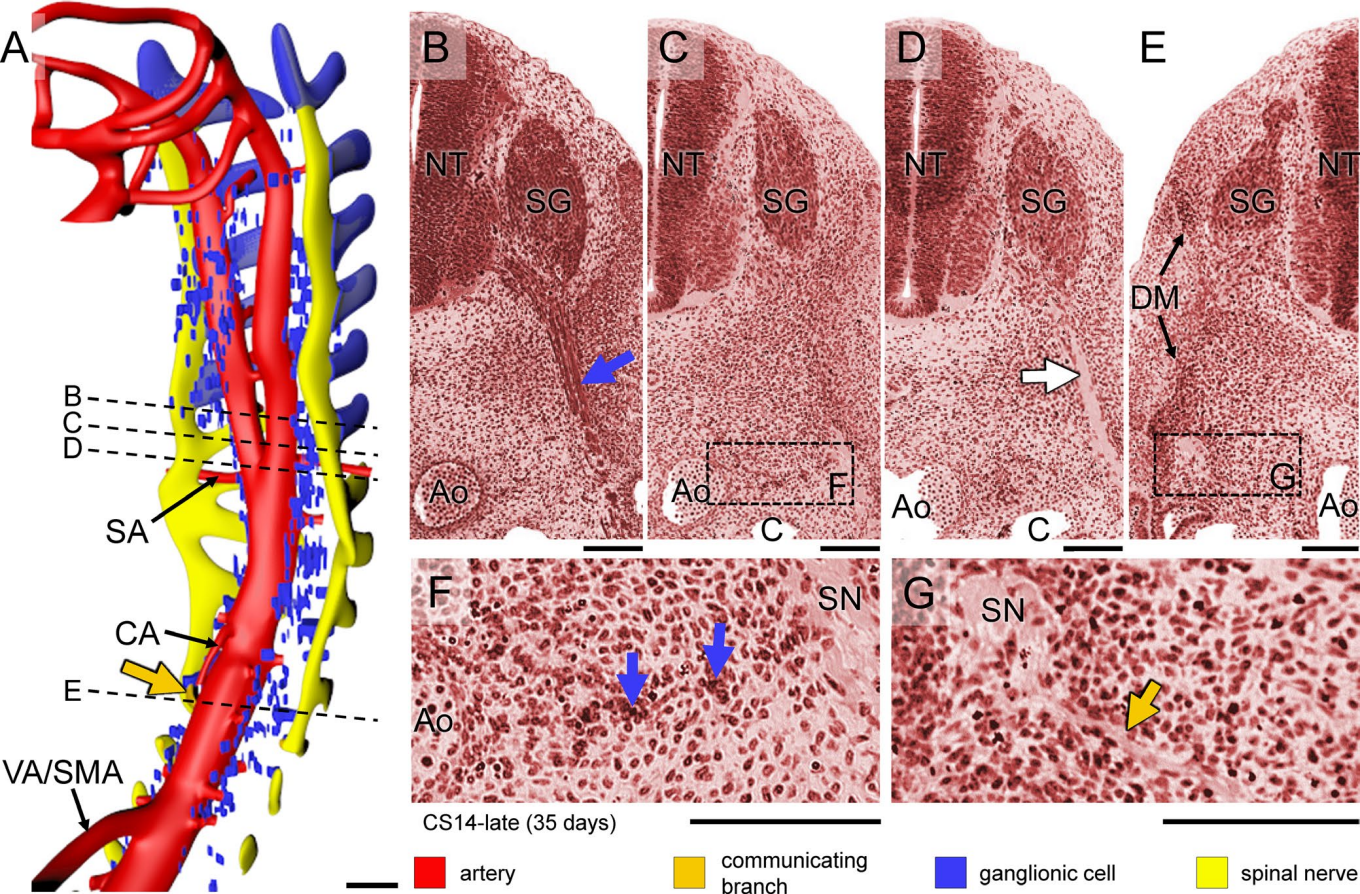
Formation of sympathetic trunks and communicating branches in a CS14‐late embryo (~35 days). Panel (a) shows the distribution of ganglionic cells with the ventral roots of spinal nerves and dorsal aorta from C1 to T5 (see also Figure S3B). Panels (b–e) show transverse sections from cranial to caudal as indicated by dotted lines in panel (a). Panels (f and g) show magnified views of the rectangles in panels (c and e), respectively. In this embryo, Schwann cell precursors cells migrate along the ventral roots of spinal nerves as intensely staining strands in the cervical region (blue cover on spinal roots in panel (a) and blue arrow in panel (b)), but not further caudally (pale nerve strand; white arrow in panel (d)). Ganglionic cells (blue arrows) are also present between the dorsal aorta (Ao) and spinal nerves (SN) in this area (panel f). Furthermore, nerve fibres extend medially following the route of ganglionic cells as communicating branches in the upper thoracic region (dark‐yellow arrow in panels [a,g]). Bars (a) = 200 µm; (b–g) = 100 µm [Colour figure can be viewed at wileyonlinelibrary.com]

The neural crest had formed and its cells were actively migrating in CS13 human embryos (~32 days of development [O'Rahilly and Müller, 2007]), but spinal nerves and their associated ganglionic cells still had to develop. Small scattered intensely basophilic cells appeared laterally to the dorsal aorta at CS14‐early (~33 days; Figure 1, blue arrows in panel c). We identified these cells as neural crest‐derived cells, because phenotypically similar cells at this developmental stage and location had previously been shown to be c‐RET‐, SOX10‐ and p75^NTR^‐positive neural crest cells (Fu *et al*., 2003, Wallace and Burns, 2005). Cells with similar staining properties as the neural crest‐derived ganglionic cells in Figure 1c transiently accumulated on the vagus nerve in CS14‐mid (~34 days; not shown), on the spinal nerves of cervical segments C1‐C6 in CS14‐late (~35 days; Figure 2a,b), and at a lower density on more caudal nerves in CS15 embryos (not shown). Spinal nerves in front of the ‘wave’ did not show such nerve‐associated cells (e.g. pale nerve fibres in Figure 2d for CS14‐late).

### 3.2 Early development of the sympathetic trunk

**Figure 3:**
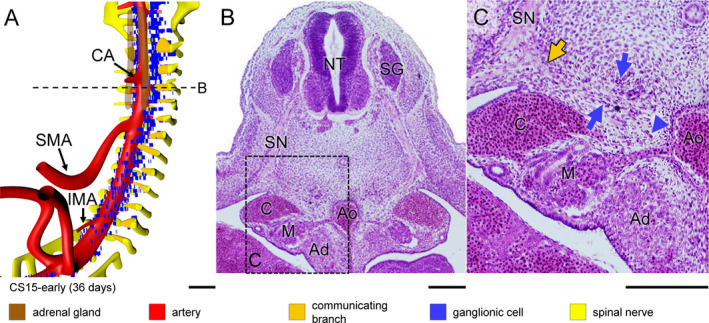
Aggregation of ganglionic cells dorsolateral to aorta in a CS15‐early embryo (~36 days). Panel (a) shows the distribution of ganglionic cells with ventral roots of spinal nerves, communicating branches, and dorsal aorta (see also Figure S3C). Panels (b and c) show a transverse section and its magnified view, respectively, as indicated by a dotted line in panel (a). Two groups of ganglionic cells are seen: aggregated sympathetic ganglia dorsolateral to the dorsal aorta, and ventrally migrating ganglionic cells (blue arrows and arrowhead in panel (c), respectively). In addition, communicating branches are present (beige arrow in panel [c]). Bars (a) = 200 µm; (b‐c) = 100 µm [Colour figure can be viewed at wileyonlinelibrary.com]

**Figure 4:**
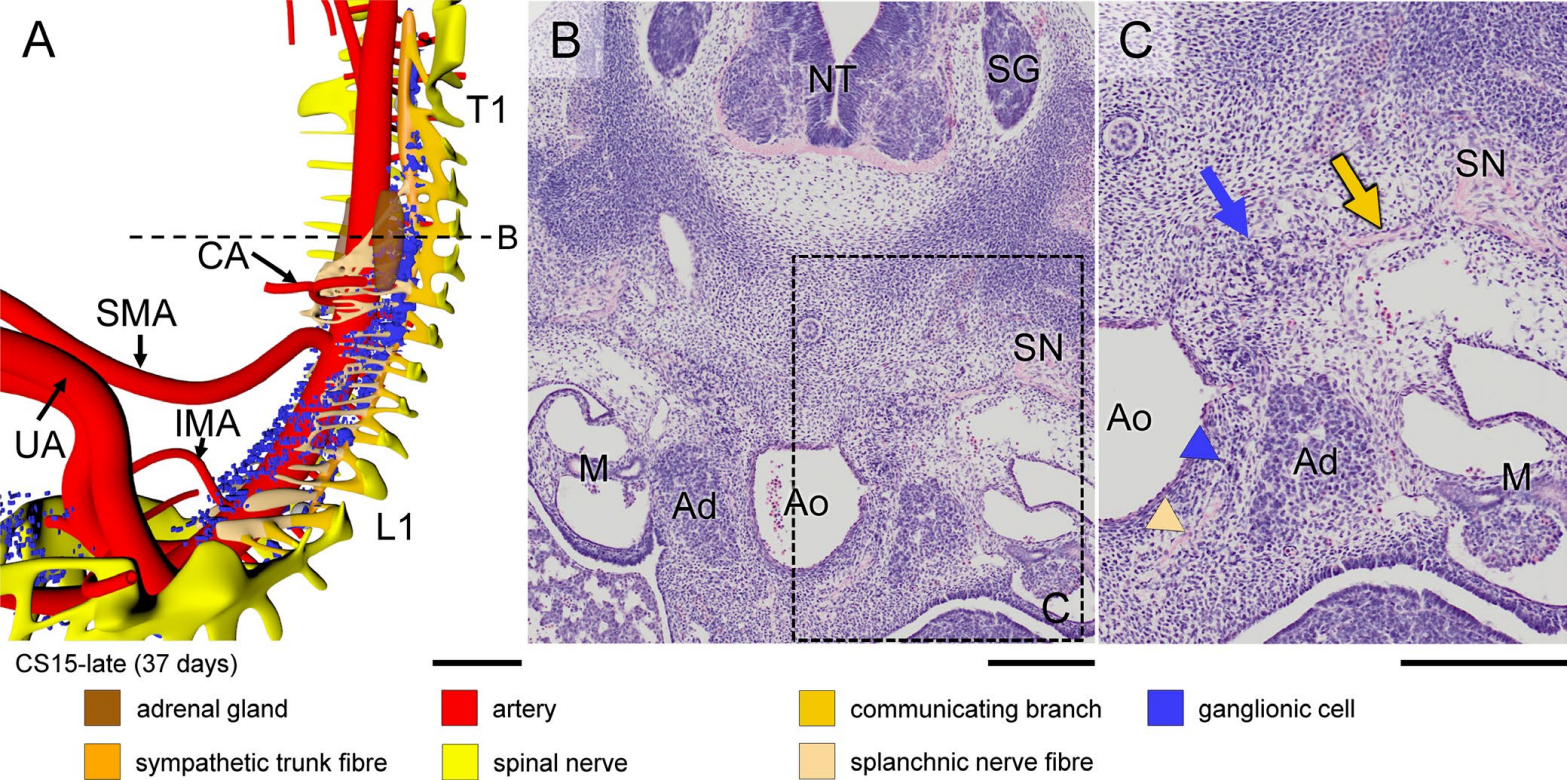
Formation of ventral (pre‐aortic) plexuses in a CS15‐late (~37 days) embryo. Panel (a) shows the distribution of ganglionic cells and nerve fibres around the aorta (see also Figure S3D). Panels (b and c) show a transverse section and its magnified view, respectively, as indicated by the dotted line in panel (a). Note communicating branch and sympathetic trunk (beige and blue arrows, respectively). Both ganglionic cells and nerve fibres (blue and beige arrowheads, respectively) migrate ventrally between the aorta and developing adrenal gland (Ad). Ganglionic cells accumulated around the roots of the CA and IMA, but not yet around the SMA (panel [a]). In addition, ventrally extending nerve fibres have formed a plexus around the CA. Bars (a) = 500 µm; (b‐c) = 200 µm [Colour figure can be viewed at wileyonlinelibrary.com]

In CS14‐late embryos (~35 days), the number of ganglionic cells lateral to the dorsal aorta had increased markedly between the mid‐cervical and lower thoracic segments. In the next few days (CS15 and CS16; 36–39 days of development), part of these still scattered ganglionic cells began to form two longitudinal columns laterally to the dorsal aorta. Furthermore, strands of ganglionic cells began to form caudal to cervical segment C3 between the entrance of spinal nerves into dermomyotomes laterally and the foregut and dorsal aorta medially (blue arrows in Figure 2f), together with tiny nerves (dark‐yellow arrows in Figure 2a,g; see also in Figure S3B). Based on this topography, we have identified the dorsolateral columns of scattered cells as the emerging sympathetic trunks and the small nerves as communicating branches. The communicating branches elongated from the spinal nerves to the sympathetic trunks at CS15‐early (~36 days; Figure 3, dark‐yellow arrow in panel c) and joined these trunks at CS15‐late (~37 days; Figure 4, dark‐yellow arrow in panel c). In the 2–3 days between CS14‐late and CS15‐late, the position of the forming sympathetic trunk changed from lateral to dorsolateral of the dorsal aorta (cf. Figure 2c,f; Figures 3 and 4b,c). The ganglionic cells of the sympathetic trunk extended caudally to thoracic level T8 at CS14‐late, lumbar level L2 at CS15‐early (~36 days), sacral level S1 at CS15‐late (~37 days) and S5 at CS16 (~39 days). The rate of caudal extension of the sympathetic trunk was, therefore, ~4 segments per day (Figures [Link], [Link], [Link]). The cell density in the clusters of the caudal‐most 5–6 segments of the sympathetic trunk rapidly increased during the first 1–2 days after they became identifiable. We will describe the development of the sympathetic trunk in more detail in a subsequent study.

### 3.3 Ventral migration of ganglionic cells

**Figure 5:**
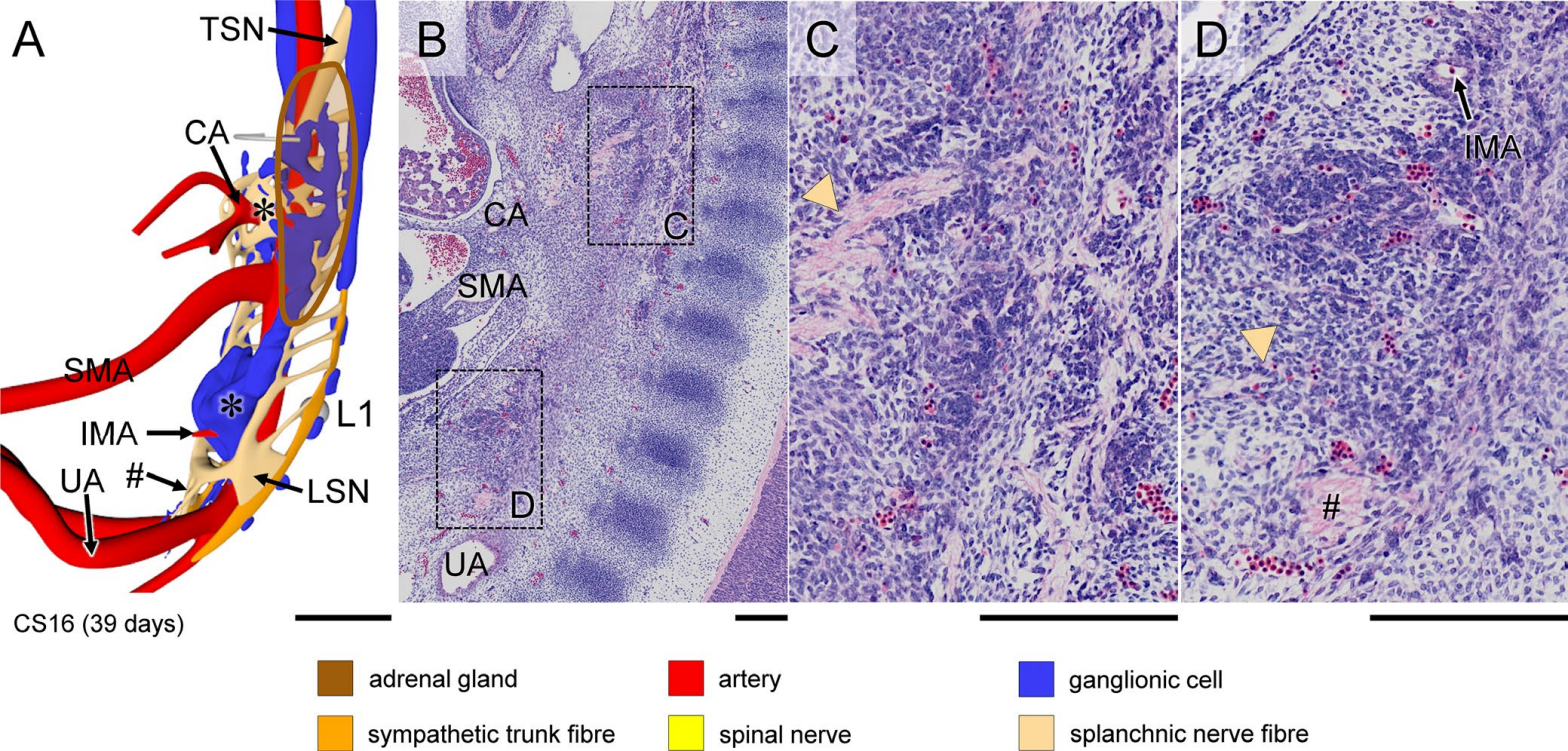
Appearance of coeliac and inferior mesenteric plexuses in a CS16 (~39 days) embryo. Panel (a) shows a right ventrolateral view of the distribution of ganglionic cells and nerve fibres, with the left adrenal gland (brown contour; see also Figure S3E). Panel (b) shows a paramedian sagittal section of the plexuses surrounding the roots of the CA and IMA (boxes), with panels (c and d) showing magnified views. The roots of both CA and IMA are surrounded by abundant ganglionic cells (indicated by asterisks in panel [a]), whereas the root of the SMA is almost devoid of ganglionic cells. Nerve fibres of the thoracic splanchnic nerves (arrowhead in panel [c]) integrate with the ganglionic cells surrounding the root of the CA. Similarly, nerve fibres of the lumbar splanchnic nerves (arrowhead in panel [d]) integrate with the ganglionic cells surrounding the root of IMA. Nerve bundles (#) from the lumbar splanchnic nerves extend caudally across the bifurcation of the umbilical arteries to form the superior hypogastric nerve. Bars (a) = 500 µm, (b‐d) = 200 µm [Colour figure can be viewed at wileyonlinelibrary.com]

**Figure 6:**
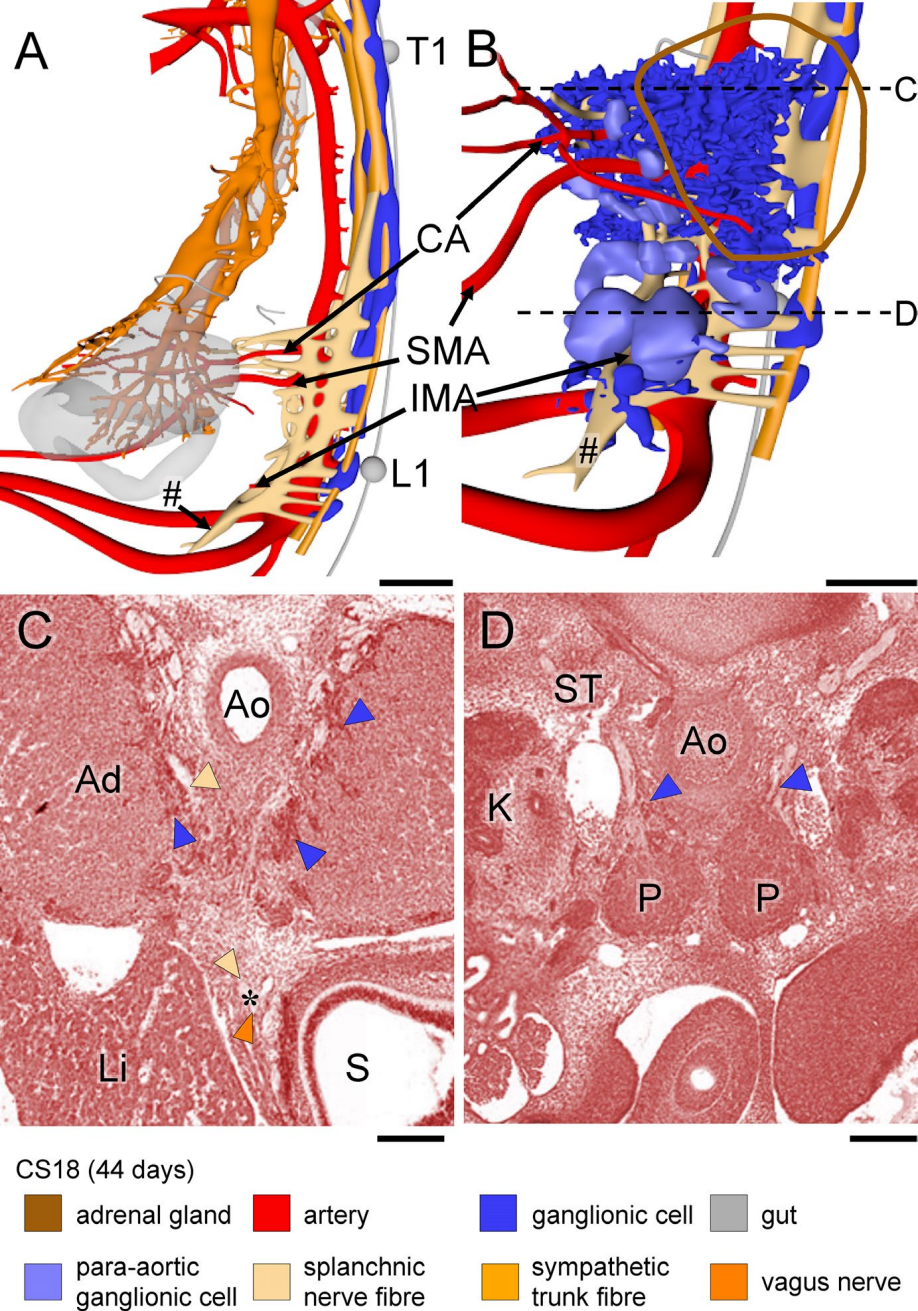
Extension of the coeliac and inferior mesenteric plexuses in a CS18 (~44 days) embryo. Panels (a and b) show side views without and with ganglionic cells in the pre‐aortic plexuses, respectively (see also Figure S4A). Ventral plexus fibres have contacted the vagal nerve plexus on the stomach (panel [a]). Panels (c and d) show transverse sections at the levels indicated by dotted lines in panel (b). Scattered ganglionic cells (blue arrowheads) surround the root of the CA and invade the adrenal cortex (AC; panels [c and d]), whereas clustered ganglionic cells form the para‐aortic bodies (P; panel [d]). A branch of the lumbar splanchnic nerves has extended caudally and passes the umbilical arteries ventrally to form the superior hypogastric nerve (# in panel [a]). Bars (a, b) = 500 µm, (c,d) = 200 µm [Colour figure can be viewed at wileyonlinelibrary.com]

**Figure 7:**
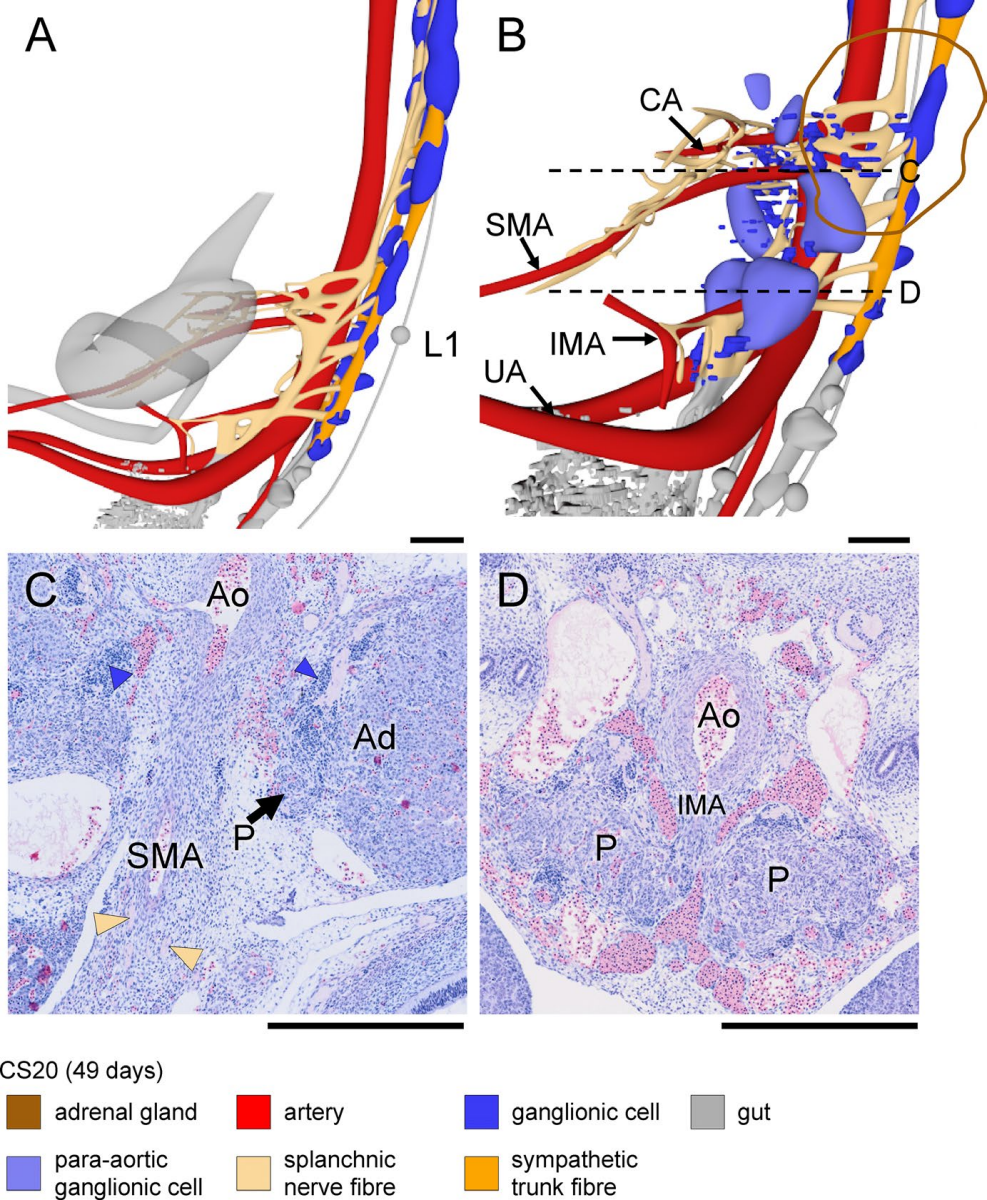
Coeliac and inferior mesenteric plexuses in a CS20 (~49 days) embryo. Panels (a and b) show side views without and with ganglionic cells in the pre‐aortic plexuses, respectively (see also Figure S4B). Panels (c and d) show transverse sections at the levels indicated in panel B. Compared to CS18 and to scattered ganglionic cells (blue arrowheads), clustered ganglionic cells have become paler (P in panels [c and d]). At the level of the IMA (panel d), para‐aortic bodies are substantially larger than at the level of the SMA (panel c). Bars = 500 µm [Colour figure can be viewed at wileyonlinelibrary.com]

Scattered ganglionic cells were also found near the oesophagus, but these cells were associated with the developing vagus nerve. We will also describe the development of the vagus nerve in a separate study. Along the medial boundary of the adrenal gland, so starting just caudal to the future diaphragm, scattered ganglionic cells remained present not only in the form of both columns of the sympathetic trunk dorsolateral to the aorta, but also as scattered cells lateral to the aorta. These lateral cells were initially (CS15‐early) most abundant between the coeliac (CA) and inferior mesenteric arteries (IMA) (Figure 3a), but caudal to the superior mesenteric artery (SMA; successor of vitelline artery), these cells then (CS15‐late, CS16) moved on towards the ventral side of the aorta (Figures 4 and 5a), so that ganglionic cells that remained associated with the adrenal cortex were mainly seen in the area between T7 and T12. At the same time (CS15‐late), nerve fibres (beige arrowhead in Figure 4c) amidst this lateral group of scattered ganglionic cells (blue arrowhead) extended ventrally and had become well‐developed entities at CS16 (~39 days of development; Figure 5a; histological details in panels [b‐d]). At CS18 (Figure 6), the number of ganglionic cells at the site of the forming adrenal medulla increased substantially (panels [b and c]), while dorsoventrally oriented nerve fibres extended further ventrally (beige arrowhead) and started to form a network around the stem of the coeliac trunk (Figure S4B). The dorsoventral fibres became subsequently (CS20) organised into nerve trunks that formed, from cranial to caudal, the greater, lesser and least splanchnic nerves, respectively, while the ventral network formed the coeliac nerve plexus (Figure 7a; Figures [Link], [Link], [Link]).

Starting also at CS16 (~39 days), a ventral plexus developed between IMA and aortic bifurcation, which, like its coeliac counterpart, was connected with the sympathetic trunk. These nerve fibres are known as the lumbar splanchnic nerves (LSN in Figure 5a). At CS16, small nerve fibres from the IMA plexus extended caudally (# in Figure 5a,d) and passed, at CS18, the root of the umbilical arteries to become the single superior hypogastric nerve (# in Figure 6a,b). This single nerve trunk began to split up into a plexus of smaller nerve fibres in CS20 embryos and is described in more detail in the accompanying article. The superior hypogastric nerve bifurcated distal to the umbilical arteries into left and right hypogastric nerves (Figure 6b). Although nerves also surrounded the IMA and the stem of the SMA, these parts of the ventral aortic plexus started to develop relatively late (CS20; ~49 days) and became well‐developed only by CS22 (~53 days).

### 3.4 Fate of para‐aortic ganglionic cells

**Figure 8:**
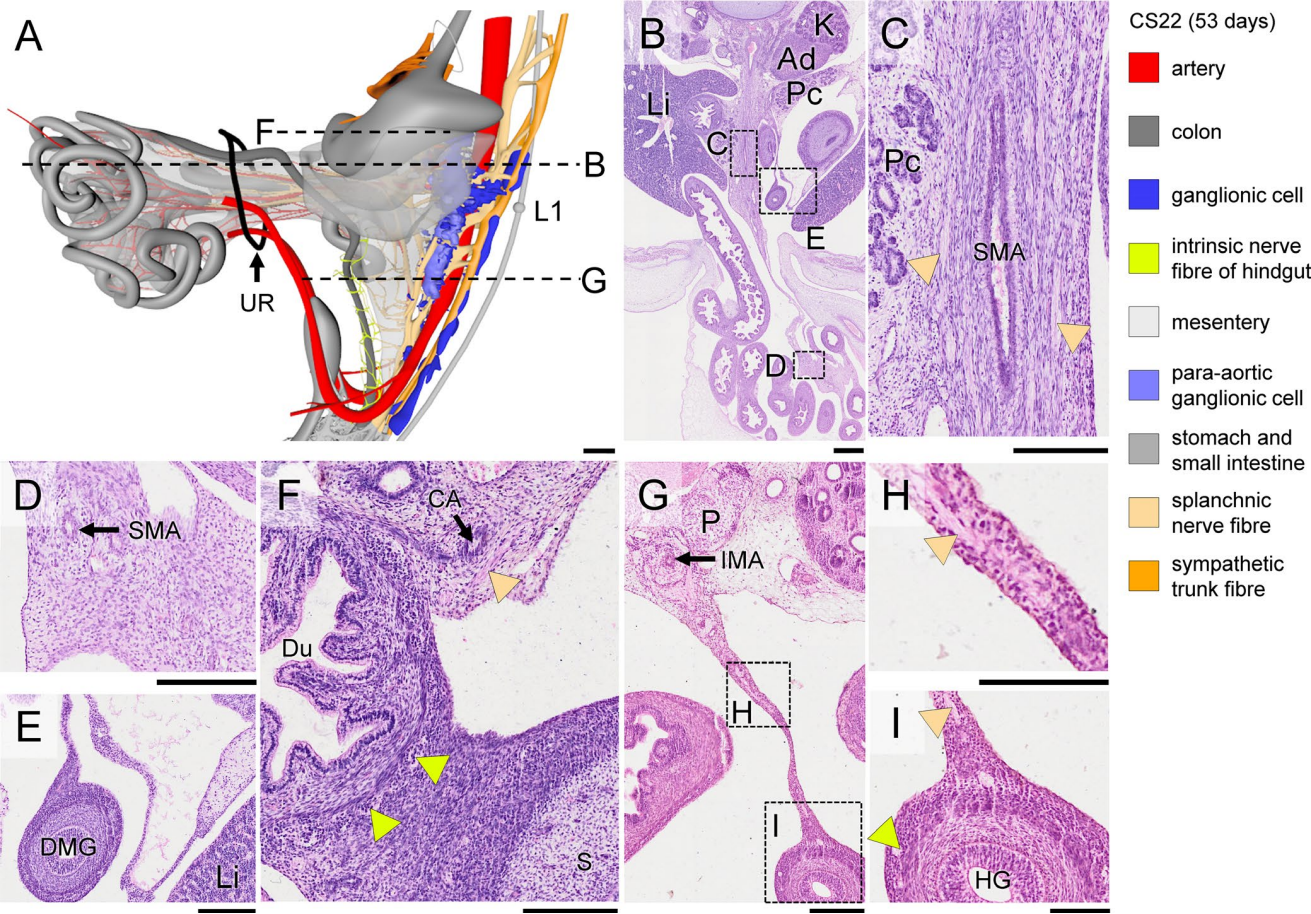
Expansion of extrinsic nerves along the superior mesenteric artery and appearance of contacts between extrinsic and intrinsic enteric nerves in a CS22 (~53 days) embryo. Panel (a) shows a side view, including the umbilical hernia of the midgut (black ring: umbilical ring; note further that the mesentery is rendered in transparent grey; see also Figure S5A). Panel (b) shows a transverse section at the level indicated in panel (a), with magnified views of the root of mesentery containing the main trunk of SMA, extra‐abdominal loops of the small intestine and colon in panels (c‐e), respectively. Panel (f) shows a transverse section of the duodenum at the level indicated in panel (a). Note numerous well‐developed intrinsic ganglia, but absence of nerve fibres in the duodenal wall, as opposed to the configuration of the hindgut (panel [i]). Panel (g) shows a transverse section of the midgut (thin mesentery) at the level indicated in panel (a), with the boxes magnified in panels (h and i). Nerve fibres extend along branches of the SMA to the intra‐abdominal small intestine and caecum (beige arrowheads in panel [c]), but not to the extra‐abdominal part of the small intestine and proximal colon (panels [d and e], respectively). In the hindgut, the extrinsic nerve fibres (beige arrowheads in panels [h and i]) have extended along the mesentery and connect with nerve fibres within the hindgut mesenchyme (light green arrowhead in panel (i). Bars (a‐d) = 500 µm, (e‐j) = 200 µm, (k‐l) = 100 µm [Colour figure can be viewed at wileyonlinelibrary.com]

The para‐aortic ganglionic cells either retained their intense staining mode or differentiated into pale‐staining cells that typically formed coherent agglomerates. The former group of cells evolved into ganglionic cells that were to become the adrenal medulla, whereas the latter transformed into the so‐called para‐aortic bodies (Coupland, 1952). The intensely staining scattered ganglionic cells predominated cranially near the CA and covered the medial side of the adrenal cortex, which they had started to penetrate with finger‐like extensions (blue arrowheads in Figure 6c). The intensely staining cells tapered off caudally (blue arrowheads in Figure 6d), where smaller aggregates were seen in association with the superior hypogastric nerve (Figure 6b). Differentiation of the para‐aortic bodies (colour coded light blue in Figures 6, 7, 8) proceeded via the formation of coherent, but still intensely staining agglomerates at CS18 (~44 days; P in Figure 6d) to the definitive pale‐staining para‐aortic bodies at CS20 (~49 days; P in Figure 7d). The pale‐staining cells predominated caudally, with the largest, dumbbell‐shaped para‐aortic body, known as Zuckerkandl's organ (Zuckerkandl, 1901), typically straddling the IMA at its cranial side, but smaller agglomerates of pale cells were present more cranially, with the uppermost agglomerate usually seen at the level of the adrenals (Figure 7b). The opposite and partially overlapping gradients between persistence of scattered, intensely staining ganglionic cells and formation of para‐aortic bodies were striking and obvious in CS18 and CS20 embryos.

### 3.5 Extension of nerves along the ventral abdominal arteries

No ganglionic cells or nerve fibres were found around any of the three abdominal arterial trunks until CS15‐late (~37 days), when nerve fibres began to surround the root of the CA (Figure 4a). Ganglionic cells followed at CS16 (~39 days) and more massively at CS18 (~44 days), concomitant with a major increase in the number of ganglionic cells at the site of the forming adrenal medulla (blue arrowheads in Figure 6c,d). Ganglionic cells surrounded the roots of the SMA and IMA with a similar timeline as described for the CA (Figure 6a,b), but nerve fibres passed the clusters of ganglionic cells (beige in Figure 7b) to extend along the stems of the SMA and IMA ~ 2 stages (CS20) later than those along the stem of the CA (CS18).

**Figure 9:**
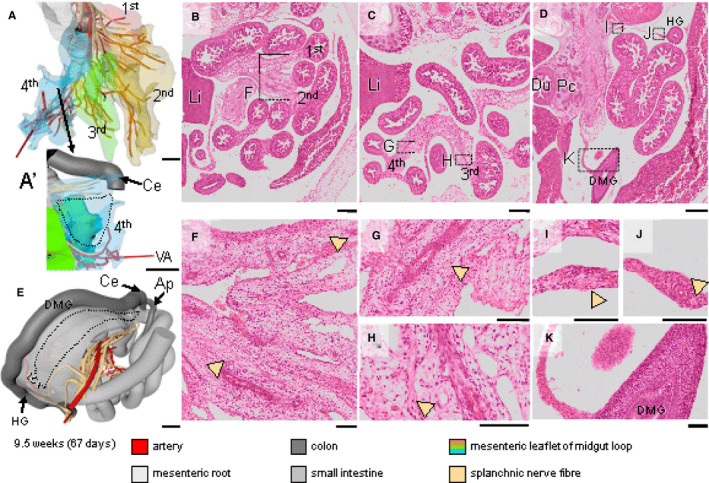
The extension of the extrinsic nerve fibres in the midgut in a 9.5 weeks embryo. Panels (a and e) show the midgut region in frontal and dorsolateral views, respectively; see also Figure S5B). The intestines have returned into the abdominal cavity. Panels (b‐d) show transverse sections of the abdominal cavity and intestinal loops, with magnified views of the boxes in panels (f‐k). The small intestinal part of the midgut has four secondary loops, each of them anchored by its own mesenterial fold (1st–4th; panel [a]; (Soffers *et al*., 2015)). The extrinsic nerve fibres along branches of the SMA have reached all midgut loops (beige arrowheads in panels (f‐h) except for the distal ileum (dotted line in panel [a']) and proximal colon (dotted line in panel [e] and panel [k]), whereas the distal colon and hindgut is innervated by nerve fibres extending along the IMA (beige arrowheads in panel [i‐j]). Bars (a,e) = 1 mm, (b‐d) = 500 µm, (f‐k) = 100 µm [Colour figure can be viewed at wileyonlinelibrary.com]

Nerves of the coeliac plexus started to extend along the main stem and branches of the CA at CS18 (~44 days) to the duodenum ventrally and to the stomach and spleen craniodorsally. These nerve branches (beige) met those of the vagal trunks (orange) on the surface of the stomach (orange‐coded nerve tree in Figure 6a and asterisk in Figure 6c). In the reconstructed embryo, the connection was best developed via the coeliac branch of the posterior vagal trunk. At CS20 (~49 days), nerve fibres also extended distally along the main trunk of the IMA and, to a lesser extent, of the SMA (Figure 7a). At CS22 (~53 days; Figure 8), the nerve fibres surrounding the CA extended to the surface of the stomach, and those surrounding the IMA to the surface of the hindgut (Figure S5A). Histologically, we established that, at this stage, both extrinsic plexuses (Figure 8c; beige arrowheads) connected to the intrinsic nerve fibres (Figure 8c,h,i; light green arrowheads) along the corresponding parts of the gut wall. Meanwhile, nerve fibres surrounding the SMA that extended within the dorsal mesentery of the midgut loop followed the main trunk of that artery to the caecum, but the nerves surrounding its other branches only reached to the umbilical ring (Figure S5A). Accordingly, the first secondary gut loop, which comprises distal duodenum and proximal jejunum and never leaves the peritoneal cavity (Soffers *et al*., 2015), had become colonised by extrinsic nerves at 8 weeks. Extrinsic innervation of the 2nd–4th secondary loops, which do herniate, developed by extension along the arterial branches of the SMA only upon the return of these loops into the peritoneal cavity during the 9th week of development (Figure 9b,c,f‐h). However, in the 9.5 weeks embryo, extrinsic nerves had not yet reached the surface of the 4th secondary loop (distal ileum containing the regressing, but still patent vitelline artery; Figure 9a’) and the colic part of the midgut between caecum and left colic artery (dotted line in Figure 9e, histological details in panels [d, k]). The extrinsic fibres, therefore, reached the caecum well before those to the distal ileum and colic part of the midgut arrived at their destination (Figure S4B).

### 3.6 Neural crest cell‐induced changes in intestinal‐wall architecture

**Figure 10:**
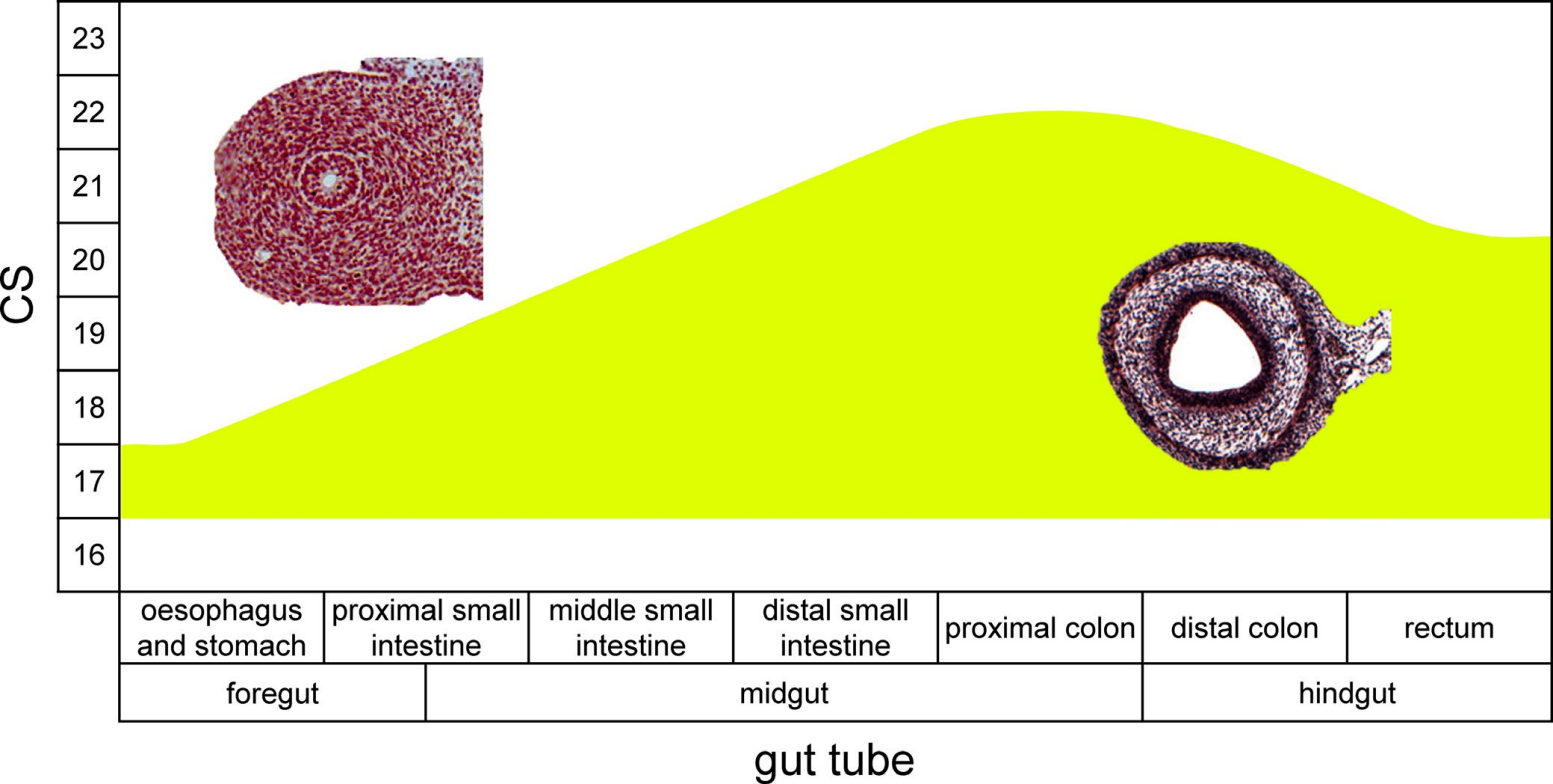
Time line of the appearance of layering in the gut wall of human embryos. The histological sections show the absence (left) and presence (right) of the layered gut wall. The layered gut wall appears first in oesophagus, stomach and proximal small intestine at CS17, in the middle portion of the small intestine at CS18, in the distal small intestine and distal hindgut (just cranial to cloaca) at CS20, and in the rest of the colon (ascending limb and hindgut) at CS22 [Colour figure can be viewed at wileyonlinelibrary.com]

Vagal neural crest‐derived cells that will form the intrinsic ENS begin to migrate through the wall of the gut at CS14 (Fu *et al*., 2003, Wallace and Burns, 2005). We noticed that the arrival of these cells changed the morphology of the intestinal wall by inducing the appearance of a layered architecture with p75^NTR^‐positive cells forming a prominent and characteristic peripheral layer (Wallace and Burns, 2005). To be able to correlate the progression of the extrinsic to that of the intrinsic enteric nervous system, we mapped the time line of the appearance of this layer (Figure 10). The external ring of cells became first visible in the foregut (oesophagus, stomach and proximal small intestine) at CS17, in the middle portion of the small intestine at CS18, in the distal small intestine and hindgut just cranial to the cloaca (Kruepunga *et al*., 2018) at CS20, and in the remaining parts of the colon (distal limb of midgut) at CS22. The delay between the passage of the migratory front of neural crest‐derived cells and the subsequent transformation of the architecture of the gut wall was 2‐3 Carnegie stages in the small intestine and ~4 Carnegie stages in the colon.

## 4 Discussion

**Figure 11:**
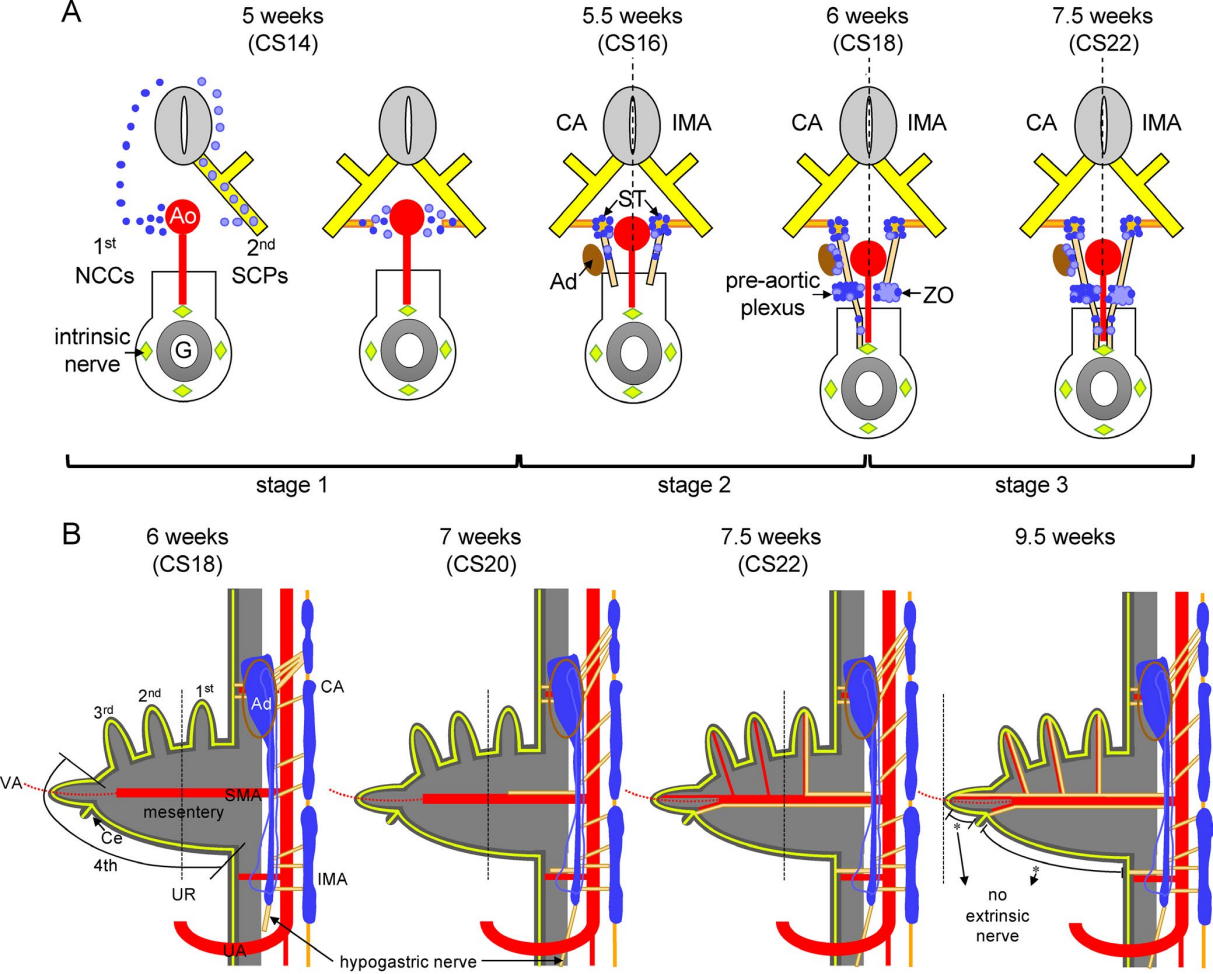
Scheme of the development of the extrinsic innervation in the thoraco‐abdominal body cavity. Panel A shows transverse views with increasing age of the embryo. Starting at 5.5 weeks, the left side of each scheme represents the level of the CA and the right side that of the IMA. At 5 weeks NCCs (blue) or SCPs (light blue) migrate through the mesenchyme or along the ventral roots of spinal nerves (yellow), respectively, to the para‐aortic region. Slightly later, nerve fibres extend medially as communicating branches (gold). At 5.5 weeks ganglionic cells either remain in the para‐aortic region to form the sympathetic trunk (ST) or migrate further ventrally. Concomitantly, nerve fibres (beige) extend ventrally to form the thoracic and lumbar splanchnic nerves (left and right part of panel, respectively). Note that the adrenal glands (Ad; brown) have formed at the level of the CA. At 6 weeks, most SCP‐derived ganglionic cells migrate towards the adrenal cortex to become the adrenal medulla or aggregate to form para‐aortic bodies at the level of the IMA. At the level of the CA, pre‐aortic nerve fibres extend further ventrally to connect to the intrinsic nerve fibres inside the stomach wall (light green diamonds). At 7.5 weeks, nerve fibres at the level of the IMA also connect to the intrinsic nerve fibres in the gut wall. Panel B shows the spatiotemporal distribution of the ganglionic cells and extrinsic nerve fibres along the ventral branches of the aorta. From 6 weeks onwards the opposing gradients of the cranially prominent adrenal medulla (blue) and the caudally prominent para‐aortic bodies (lighter blue) form. Preganglionic nerve fibres form the thoracic and lumbar splanchnic nerves (beige), while periarterial extrinsic nerve fibres extend and connect with the intrinsic nerve fibres (light green lines). Lumbar nerves extend caudally across the aortic bifurcation into the lesser pelvis (arrow). Nerve fibres surrounding the SMA and IMA extend through the mesentery (grey) to connect to the intrinsic fibres of the 1st secondary intestinal loop, caecum and hindgut at 8 weeks, and to those of the 2nd and 3rd secondary intestinal loops at 9.5 weeks. Note, however, that extrinsic nerves have not yet appeared in the mesentery of the colon at this stage (asterisks) [Colour figure can be viewed at wileyonlinelibrary.com]

The present study shows that extrinsic innervation of the gut becomes established between ~5 and ~9.5 weeks of development. We distinguished three stages (Figure 11): migration of neural crest cells (NCCs) to the para‐aortic area (5th week; CS14 and CS15); migration of ganglionic cells and nerve fibres from the para‐ to the pre‐aortic areas and stems of the abdominal arteries, in particular the coeliac (CA) and inferiors mesenteric (IMA) arteries (6th week; CS16‐18); and finally the extension of mainly nerve fibres along the main arteries and their connection with the intrinsic ENS (7th–9th week). At 9.5 weeks, however, extrinsic innervation of the colon was not yet complete.

### 4.1 Early development of the extrinsic ENS

The extrinsic ENS and sympatho‐adrenal lineage share an early stage in development. Part of the NCCs that exit the closing neural tube migrate between the neural tube and the dermomyotome towards the dorsal aorta (the so‐called medioventral pathway; NCCs in Figure 11a) (Serbedzija *et al*., 1990). Schwann cell precursors (SCPs), which are regarded as neural crest‐derived pluripotent cells that appear slightly later (Petersen and Adameyko, 2017), migrate in contact with the ventral roots of the spinal nerves (SCPs in Figure 11a). They serve as precursors for the adrenal medulla (Furlan *et al*., 2017), para‐aortic bodies (a.k.a. chromaffin paraganglia) (Kastriti *et al*., 2019) and part of the intrinsic gut ganglia (Uesaka *et al*., 2015). We studied 12 CS14 and CS15 embryos, in which we found SCPs transiently present along the vagus nerve (CS14‐mid), cervical spinal nerves (C1‐C6; CS14‐late), and more caudal spinal nerves (CS15). The presence of SCPs on the nerve trunks was short (~1 day) and their density on the spinal nerves highest cranially. Migration of both groups of NCCs towards the dorsal aorta is directed by chemoattractive factors that include CXCL12 (SDF1) and NRG1 produced by the dorsal aorta, and GDNF and complement component 3A (C3A) produced by gut mesenchyme (Wright and Snider, 1996, Vega‐Lopez *et al*., 2017). The many simultaneously active chemoattractants may facilitate a more precise final location of the migrating cells (Dyson *et al*., 2018).

### 4.2 Formation of adrenal medulla and para‐aortic bodies

Ventral migration of paravertebral neural crest‐derived ganglionic cells was first seen at CS15‐early and was most prominent during CS16 and at all stages only observed at and caudal to the developing adrenals. The adrenals were first identifiable between C7 and T5 (CS15‐early), ‘descended’ to T4‐T9 during CS15‐late and CS16, to end up between T11‐L3 at and after CS20 (Hikspoors *et al*., 2015). The cell bodies of the preganglionic nerves to the adrenal medulla locate to the intermediolateral horn of segments T1‐L1, whereas the postganglionic nerves originate in sympathetic ganglia at levels T4‐12 (Kesse *et al*., 1988), suggesting that medullar cells become associated with the cortex early during its descent. The location, cellularity and fate of these SCP‐derived ganglionic cells exhibited pronounced and opposite cranial‐to‐caudal gradients, with cells lateral to the aorta predominating cranially as adrenal medulla and cells ventral to the aorta caudally as para‐aortic bodies (Coupland, 1952). The fate of the cranial and caudal ganglionic cells also reflected the observed craniocaudal gradient in cell aggregation: the cells forming the future adrenal medulla retained their scattered distribution, whereas the para‐aortic bodies formed tightly packed, pale‐staining cell agglomerates at CS18 (‘6 weeks’ in Figure 11a). The largest para‐aortic body, which straddles the aorta cranial to the IMA, is known as Zuckerkandl's organ. Zuckerkandl's organ is largest in infants and regresses in toddlers (Zuckerkandl, 1901; Coupland, 1954). This topographic gradient was also seen in reconstructions of mouse embryos (Furlan *et al*., 2017) but, as far as we are aware, the mechanism that underlies these opposing gradients is still unknown. In addition, the dorsoventrally oriented nerve fibres were most numerous cranially. In all likelihood, these nerve fibres provide a matrix for SCPs to migrate, since ~80% of the chromaffin cells of the adrenal medulla and pre‐aortic paraganglia are formed from such SCPs (Furlan *et al*., 2017; Kastriti *et al*., 2019).

### 4.3 Formation of pre‐aortic plexuses and extension of nerves along the ventral abdominal arteries

Scattered neural crest‐derived cells and nerve fibres form the pre‐aortic nerve plexuses. The plexuses surrounding the CA became identifiable at CS16 and further extended along the CA beyond the cluster of ganglionic cells at CS18, whereas that surrounding the IMA and stem of SMA acquired a similar stage of development at CS20. Accordingly, nerve fibres started to extend along the CA and IMA towards the gut tube well before those along the branches of SMA (Figure 11b). A subsequent striking feature was the apparent blockade of nerve extension along the branches of the SMA that passed through the umbilical ring to perfuse the midgut loops in the umbilical hernia. As a result, the extrinsic nerves had reached the gut wall of the non‐herniating 1st secondary loop of the small intestine (duodenum and proximal jejunum; (Soffers *et al*., 2015)) and the hindgut already at CS22 (7.5 weeks), whereas the herniating 2nd to 4th secondary loops of the gut became innervated by extrinsic nerves only after their return into the peritoneal cavity between 9 and 9.5 weeks of development (Soffers *et al*., 2015). Consequently, the walls of the distal ileum and proximal colon were still not innervated at 9.5 weeks of development (Figure 11b). To the best of our knowledge, this regional difference in the development of the extrinsic innervation of the gut has not yet been reported. The delay in innervation may correspond with the rapid growth of the midgut resulting in the formation of loops (Soffers *et al*., 2015; Ueda *et al*., 2016). In agreement, the ganglionic cells of the intrinsic ENS begin to form the myenteric plexus at ~CS22 (Okamoto and Ueda, 1967, Fu *et al*., 2004, Wallace and Burns, 2005), just prior to intestinal return and resolution of the hernia (Soffers *et al*., 2015; Nagata *et al*., 2019). Furthermore, it has been reported, however, that the mitotic index in the ileocaecal region of 11–12 weeks old foetuses was still ~2‐fold higher than in the oesophagus or hindgut (Vaos, 1989), while enteric neurons were less developed in the ileum than in adjacent parts of the gut (Tam, 1986). Since cell‐cycle withdrawal usually precedes neuronal differentiation in the gut (Pham *et al*., 1991; Chalazonitis *et al*., 2008; Bergner *et al*., 2014), these findings could point at more growth and less differentiation in the herniating part of the midgut.

### 4.4 Timeline of intrinsic and extrinsic innervation of the gut

The timeline of the development of the intrinsic autonomic innervation in experimental animals is known in far greater detail than that of the extrinsic autonomic innervation. In mouse embryos, the colonisation of the wall of the gut by caudal vagal neural crest‐derived cells is reported to proceed as a unidirectional craniocaudal wave that begins at ED9.5 (Theiler's stage (TS) 15; ~CS13) in the foregut and reaches the proximal small intestine at ED10.5 (TS17; CS14) (Erickson *et al*., 2014, Hatch and Mukouyama, 2015) and the terminal ileum at ED11.0 (TS18; ~CS15) (Young *et al*., 1998, Druckenbrod and Epstein, 2005, Anderson *et al*., 2006). Here, the caudal‐ward progression temporarily slows while the NCCs bypass the caecum by taking a transmesenteric route to migrate from the proximal (ileal) to the distal (colonic) limb of the then forming primary intestinal loop (Soffers *et al*., 2015). Caudal migration resumes at ED12.0 (TS 20; CS17) and reaches the hindgut at ED14.0 (TS22; ~CS21) (Young *et al*., 1998, Druckenbrod and Epstein, 2005, Anderson *et al*., 2006, Erickson *et al*., 2014). The intrinsic NCCs may (Hatch and Mukouyama, 2015) or may not (Delalande *et al*., 2014) follow the advancing front of the developing enteric capillary plexus. The generally accepted view is that the extension of extrinsic nerves along the intestinal arteries follows the intrinsic innervation in mice with a delay of ~2 days (~3 Carnegie stages) (Erickson *et al*., 2014, Hatch and Mukouyama, 2015, Uesaka *et al*., 2016), but has only been documented in detail in the proximal intestine (Hatch and Mukouyama, 2015).

The reported migration of NCCs through the human gut progresses at a similar rate (expressed per developmental stage) as that in mice with a delay of ~1 Carnegie stage (Okamoto and Ueda, 1967, Fu *et al*., 2004, Wallace and Burns, 2005). This delay can probably be ascribed to a lower sensitivity of the visualisation methods for NCCs in humans. In humans, the transformation of the amorphous gut mesenchyme into the layered architecture of the intestinal wall followed the passage of the wave front of NCCs by 2–3 Carnegie stages in the small intestine and ~4 stages in the colon (Figure 10). We have compared the temporal progress of the transformation of the intestinal wall in human embryos with 22 conventionally stained mouse embryos between ED11 and ED15 (~CS15‐~CS22) that were sectioned transversely (8), sagittally (7) or frontally (7). Apart from a slower development of the mural architecture in the stomach and especially the proximal duodenum in mice, the findings were comparable, with a delay of 3–4 Carnegie stages between the passage of the wavefront of neural crest‐derived cells and the transformation of the gut wall. Unfortunately, the developmental change in architecture of the midgut wall was too coarse a parameter to allow detection of a migratory delay of the intrinsic ganglionic cells at the ileocaecal junction in human embryos.

### 4.5 Region‐specific developmental pattern of the extrinsic innervation

The extrinsic innervation in the thoraco‐abdominal cavity can be divided into three subregions that follow the three main ventral branches of the dorsal aorta. Of these, the development of the autonomic plexus surrounding the root of the CA is best studied because of the presence of the developing adrenal glands (Saito and Takahashi, 2015, Furlan *et al*., 2017). In this region, NCCs migrate to the para‐aortic region to form sympathetic ganglia, followed by continued migration of SCP‐derived ganglionic cells along spinal nerve fibres, communicating branches and nerves forming the pre‐aortic plexuses (Figure 11). SCPs then migrate laterally to the developing adrenal cortex to become the chromaffin cells of the adrenal medulla (Furlan *et al*., 2017). In contrast to the ganglionic cells, which remain largely confined to the root of the CA, the nerve fibres extend further ventrally along the branches of the CA starting at CS16 (39 days) and begin to connect to the intrinsic nerve fibres at CS18 on the stomach (44 days; Figure 11a) (Fu *et al*., 2004, Wallace and Burns, 2005). The coeliac plexus is further characterised by an extensive sympathetic innervation via the greater (segments T5‐T9), lesser (segments T10, T11) and least (T12) thoracic splanchnic nerves, which become first identifiable at CS15‐late (~37 days). Interestingly, the segments from which the splanchnic nerves derive are persisting landmarks of the ‘descent’ of the coeliac and superior mesenteric arteries and adrenals. In addition to the splanchnic nerves, the vagal nerve contacts the coeliac plexus, starting with the posterior vagal trunk at CS18.

The development of the inferior mesenteric plexus follows the same general pattern as the coeliac plexus with a few notable differences, which correspond to the differentiation of the SCP‐derived cells into para‐aortic bodies rather than the adrenal medulla (Kastriti *et al*., 2019). As mentioned in the previous section, the region of the adrenal medullary cluster of cells gradually changes into one with both scattered neural cells and more tightly packed cells in the para‐aortic bodies (Coupland, 1952). The extrinsic nerves that migrate along the IMA take around 1 week [CS20 (49 days)–CS22 (53 days)] to connect to the local intrinsic nerve plexus. The sympathetic lumbar splanchnic nerves that innervate the inferior mesenteric plexus originate in segments L1‐L3 and differ from the thoracic splanchnic nerves mainly by their minimal segmental ‘descent’ (Figure 11).

## 5 CONCLUSION

The developmental patterns of the extrinsic innervation are repeated in the three subregions of the main ventral aortic branches, but with some region‐specific features. These features include the formation of adrenal medulla near the CA, the concentration of para‐aortic bodies near the IMA, and the delay in extrinsic innervation in the herniated parts of the midgut.

## CONFLICT OF INTERESTS

The authors declare that they have no competing interests.

## AUTHOR CONTRIBUTIONS

N.K. was responsible for data collection, analysis and visualisation and wrote the manuscript. J.H. and C.H. participated in data collection, analysis and interpretation. G.M. and N.K. were responsible for processing all Amira reconstructions in Cinema4D. S.E.K. participated in data analysis and interpretation, provided guidance and edited the manuscript. W.H.L. conceived the study, provided guidance, assisted with data interpretation, and preparation of the manuscript.

## Supporting information

Figure S1

Figure S2

Figure S3

Figure S4

Figure S5

Legends
