# Supplementary material for: Development of extrinsic innervation in the abdominal intestines of human embryos
Source: J Anat. 2020 Jun 29;237(4):655–71. doi: 10.1111/joa.13230 (PMC7495293; doi:10.1111/joa.13230)
Supplement: Supplementary file 3 — Figure S3 [file JOA-237-655-s003.pdf]

Development of extrinsic innervation in the abdominal intestines of human embryos

Nutmethee Kruepunga[1,2], Jill P.J.M. Hikspoors[1], Cindy J.M. Hülsman[1], Greet M.C. Mommen[1], S. Eleonore Köhler[1], Wouter H. Lamers[1*,3]

Supplemental Figure 3: Interactive 3D pdfs of the extrinsic innervation in the thoracoabdominal cavity from CS14 – CS16 embryos. Download the PDF-file (3D PDF can be opened on any computer as long as it contains Adobe PDF reader).

The 3D PDF becomes activated after 'clicking' with the mouse on the embryo. A toolbar appears at the top of the screen that includes the option 'model tree'. The model tree displays a material list of structures in the upper box and preset viewing options in the lower box. The list of visible structures can be modified by marking or unmarking a structure.

Furthermore, a structure can be rendered transparent by selecting that option from the dropdown menu after selecting the structure with the right mouse button. To manipulate the reconstruction, press the left mouse button to rotate it, the scroll button to zoom in or out, and the left and right mouse buttons simultaneously to move the embryo across the screen. The slicer button in the toolbar allows cross sections to be made. The plane of section can be adjusted with the offset and tilt options. To switch between reconstructions use the up or down arrows in the toolbar at the top.

The colour code is identical to that in the Figures and all structures are listed by name in the 'model tree'.

CS14-early (33 days)

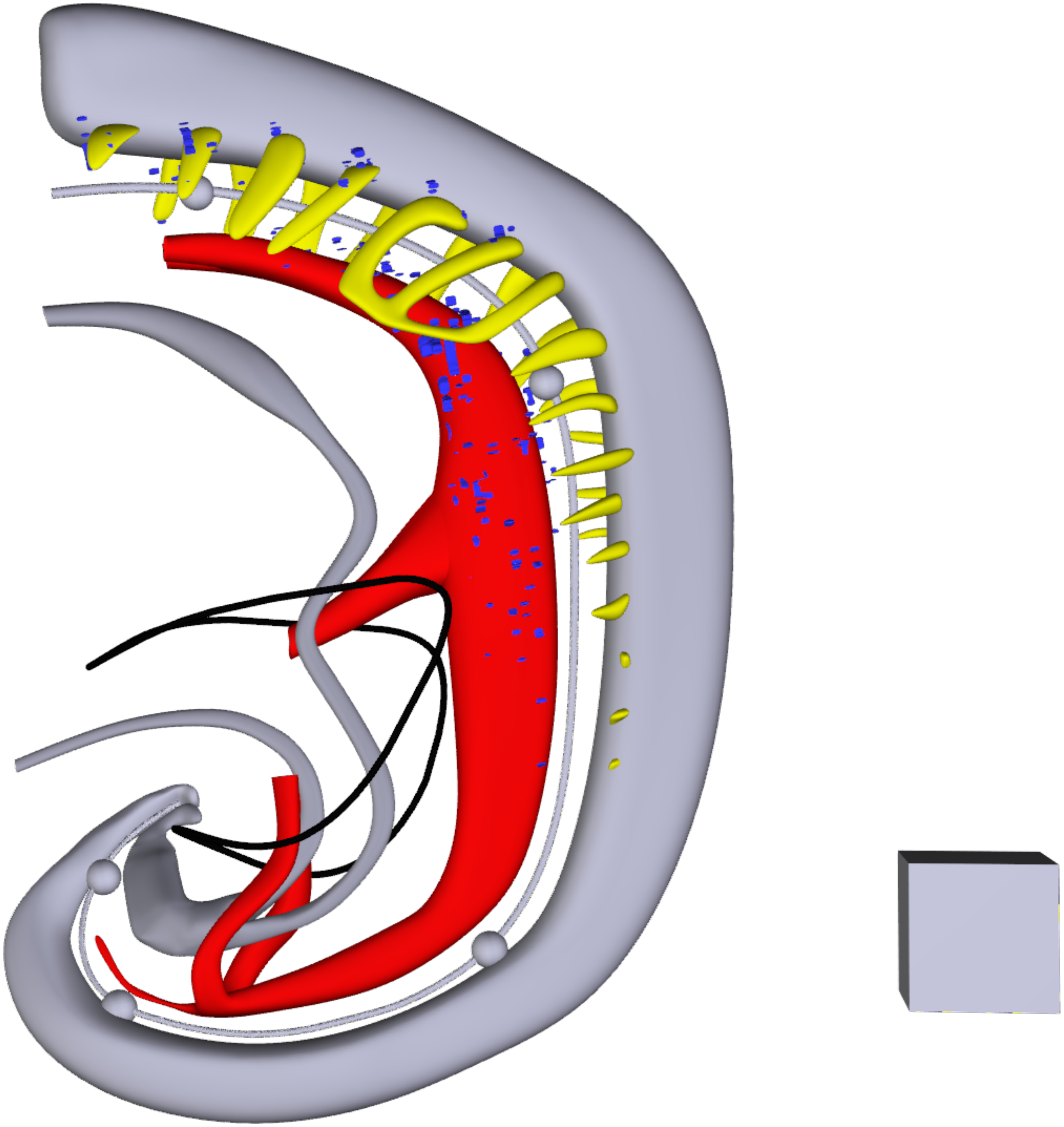

Supplemental Figure 3A

CS14-late (35 days)

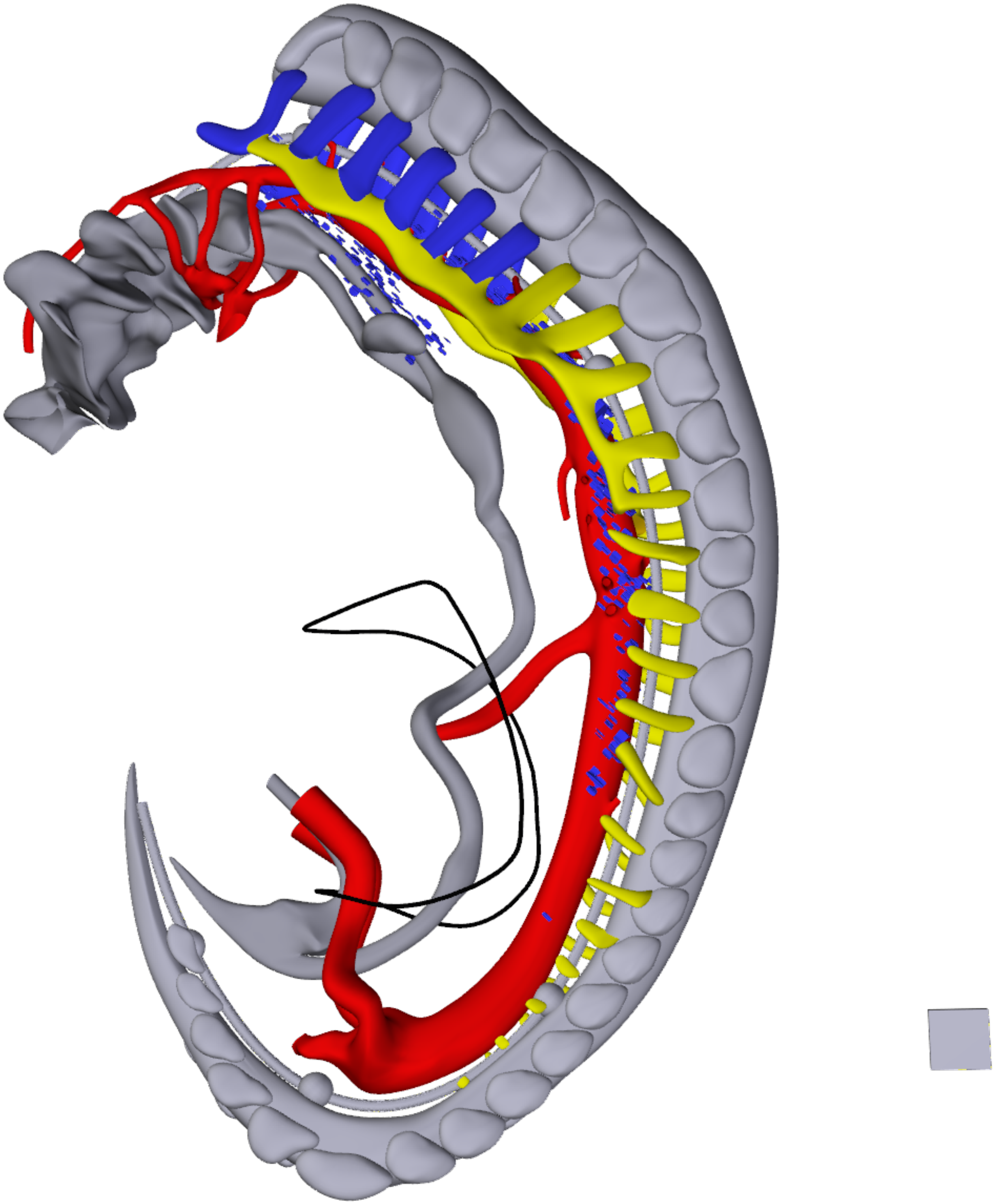

CS15-early (36 days)

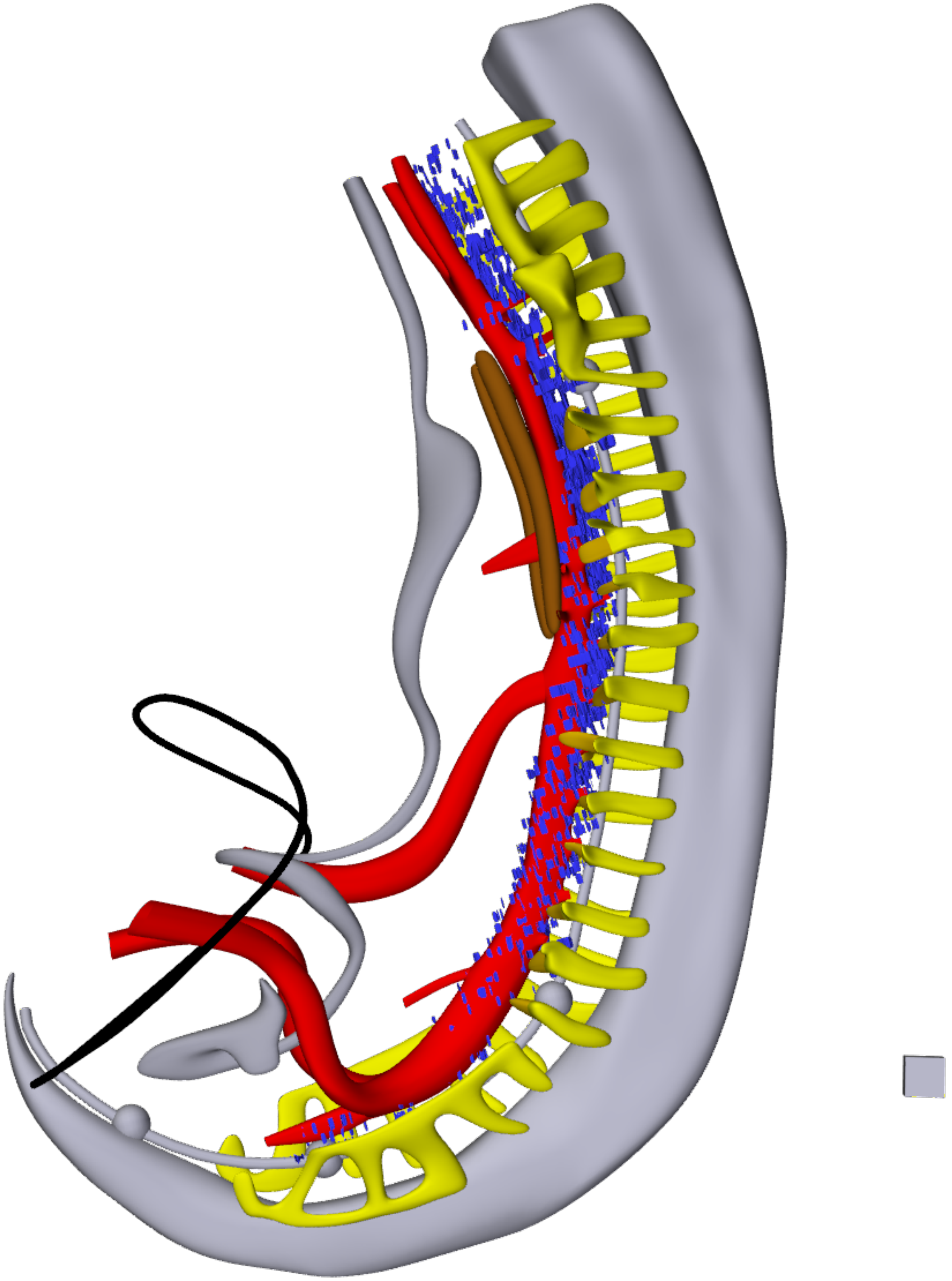

Supplemental Figure 3C

CS15-late (37 days)

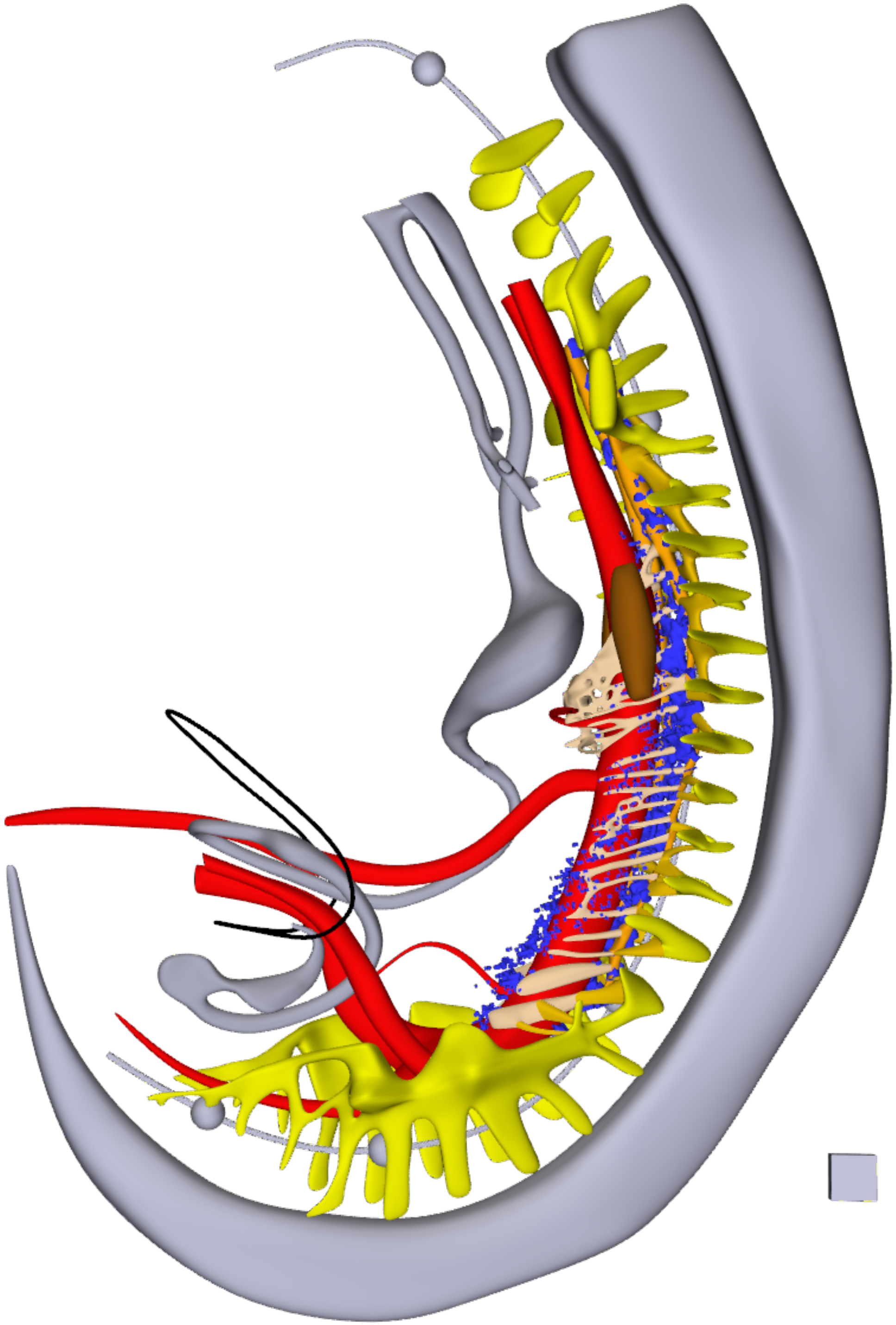

CS16 (39 days)

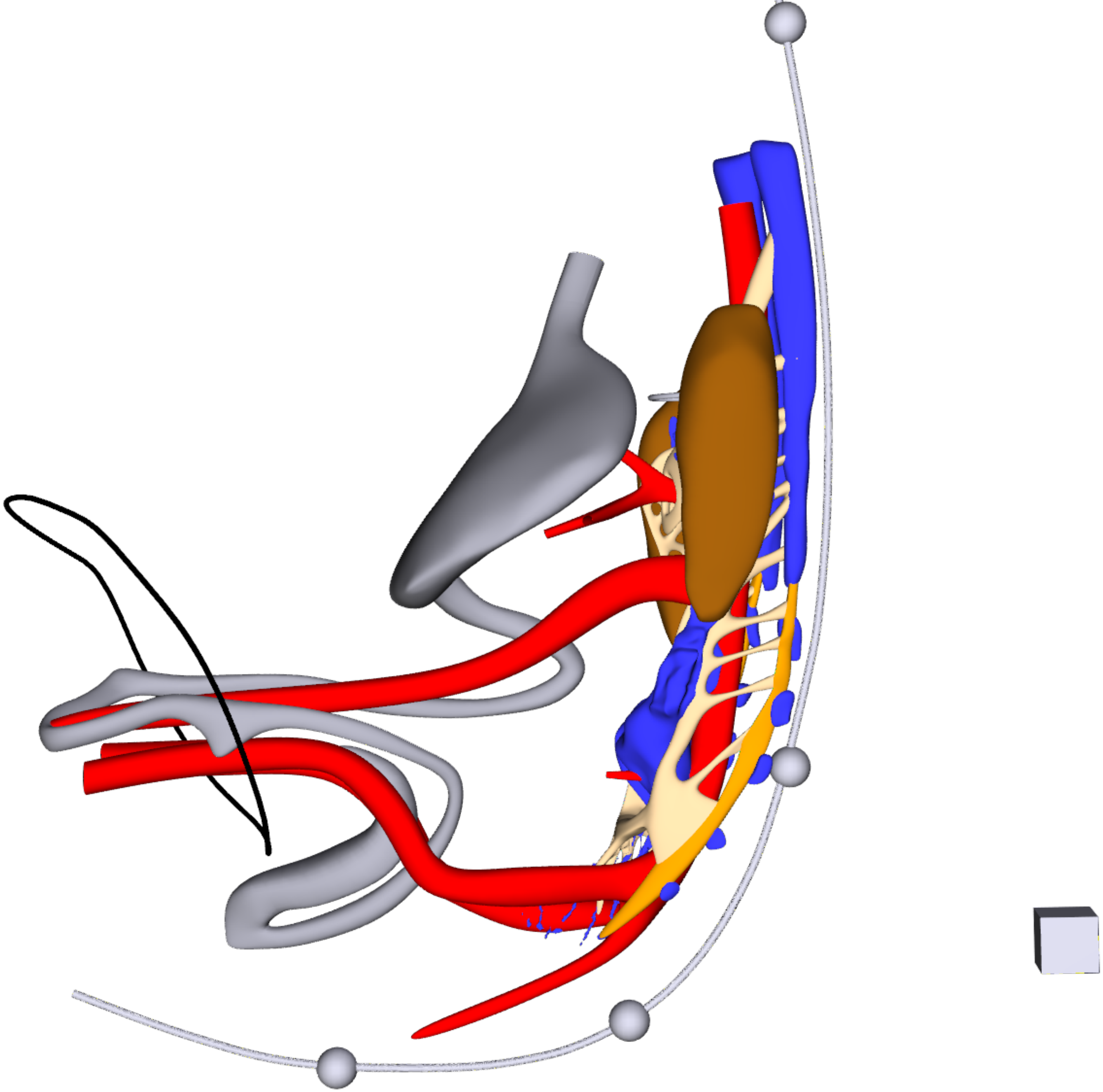

Supplemental Figure 3E
