# Supplementary material for: Development of extrinsic innervation in the abdominal intestines of human embryos
Source: J Anat. 2020 Jun 29;237(4):655–71. doi: 10.1111/joa.13230 (PMC7495293; doi:10.1111/joa.13230)
Supplement: Supplementary file 6 — Legends [file JOA-237-655-s006.docx]

**Supplemental Figure legends**

**Supplemental Figure 1:** Correlation of Carnegie stages of human embryos with days of embryonic development in mouse ([Buckingham et al., 2005](#_ENREF_4), [Krishnan et al., 2014](#_ENREF_33)) or Hamilton-Hamburger stages in chicken embryos ([Kirby, 2007](#_ENREF_32)).

**Supplemental Figure 2:** Brief procedure of 3D analysis and rendering.

**Supplemental Figure 3:** Interactive 3D PDFs of the extrinsic innervation in the thoracoabdominal cavity of CS14 – CS16 embryos.

**Supplemental Figure 4:** Interactive 3D PDFs of the extrinsic innervation in the thoracoabdominal cavity of CS18 – CS20 embryos.

**Supplemental Figure 5:** Interactive 3D PDFs of the extrinsic innervation in the thoracoabdominal cavity of CS22 – 9.5 weeks embryo and foetus.
